# Design and Function Classification Study of Multi-Bin 3D Printing Nozzle for Foam Concrete with Enhanced Performance

**DOI:** 10.3390/ma19214532

**Published:** 2026-10-28

**Authors:** Yibo Wang, Jingyi Liu, Lin Lin, Kun Yang, Min Liu, Huanshuai Zhao, Yusi Zhang, Qinglei Sun, Shiyu Ma

**Affiliations:** 1 School of Mechanical Engineering and Automation, Liaoning University of Technology, Jinzhou 121000, China; ljy2315979245@163.com (J.L.);; 2 School of Civil and Architectural Engineering, Liaoning University of Technology, Jinzhou 121000, China; 3 School of Intelligent Manufacturing, Liaoning Vocational University of Technology, Jinzhou 121000, China; 4 Key Laboratory of Digital Design and Process Simulation of Intelligent Equipment, Tangshan University, Tangshan 063000, China; 5 Institute for Advanced Marine Research, China University of Geosciences, Guangzhou 511462, China; 6 State Key Laboratory of Advanced Glass Materials, CNBM Research Institute for Advanced Glass Materials Group Co., Ltd., Bengbu 233010, China

**Keywords:** multi-bin 3D printing nozzle, functionally graded foam concrete, CFD simulation optimization, mechanical properties

## Abstract

In response to the urgent demand for lightweight and thermal insulation building materials in remote island and reef infrastructure under harsh marine environments, this paper designs a new multi-bin functional graded 3D printing nozzle and relies on the optimal base material to prepare functionally graded foam concrete. The mechanical and impact resistance properties of the layered gradient foam concrete are systematically explored. The developed nozzle consists of 4 independent auxiliary bins and 1 main bin and is equipped with a blade-type spiral structure and conical nozzle. The parameters are optimized through CFD flow field simulation, and the pitch of the auxiliary screw is set at 15 mm, the pitch of the main screw at 30 mm, and the main shaft speed at 40 r/min. This structure can achieve independent transportation of multi-component slurries and avoid material mixing. Three base materials with fiber reinforcement, high-density aggregates, and porous insulation are formulated, and the O1-O2-O3 three-layer gradient arrangement scheme can simultaneously achieve the synergistic improvement of mechanical properties and pore-structure-based thermal insulation potential. Compared with homogeneous foam concrete, the optimal graded specimen has a 60% increase in compressive strength, a 100% increase in flexural strength, and, in the specific H1-H2 comparison for which quantitative drop-hammer data were obtained, the graded specimen required more repeated impacts to first cracking and to failure than the homogeneous control, indicating improved resistance to repeated impact loading. The complete set of printing equipment and gradient composite materials can provide a preliminary technical basis for further development of intelligent on-site construction on islands.

## 1. Introduction

Traditional cast-in-place concrete construction relies on formwork support systems and manual layered pouring methods, often characterized by cumbersome procedures and excessive labor demands. This conventional approach demonstrates poor economic efficiency in constructing irregular structural components and struggles to meet the requirements of scattered infrastructure projects in remote offshore islands [1]. Concrete 3D printing, as a novel mold-free layer-by-layer deposition technology, has emerged as an innovative construction solution for both nearshore and offshore engineering applications due to its exceptional flexibility and production efficiency [2]. Island and reef environments endure prolonged exposure to high-humidity, high-salinity corrosive conditions with limited material transportation capabilities. These harsh operating conditions prevent traditional homogeneous foam concrete from simultaneously achieving multiple performance requirements—including lightweight weight, high strength, crack resistance, and thermal insulation—while the inherent trade-off between material density and mechanical properties significantly restricts its large-scale application [3]. Functional gradient foam concrete addresses these limitations by precisely controlling fiber content, aggregate composition, and micro-pore structure along the structural thickness direction, making it an excellent green prefabricated material specifically tailored for island engineering projects [4].

Extensive research has been conducted domestically and internationally on 3D-printed foam concrete and basalt fiber modification technologies. Falliano et al. [5] compared the performance differences between traditional cast and 3D-printed foam concrete, demonstrating that mixing rate critically determines the material’s internal porosity structure and macroscopic mechanical strength. Alghamdi et al. [6] developed a geopolymer-based 3D-printed foam concrete with a thermal conductivity of 0.15–0.25 W/(m·K), achieving optimal balance between lightweight properties and thermal insulation. Pasupathy et al. [7] improved the printability of fresh mixtures by incorporating lightweight aggregates, significantly reducing vertical deformation during component deposition. Hambach et al. [8] found that the oriented arrangement of short-cut basalt fibers during extrusion significantly enhances mortar toughness, though few studies have explored the performance mechanisms of fiber gradient distribution. Wang Li et al. [9] developed basalt sand-modified printed concrete, though their work focused solely on homogeneous formulations without gradient design. The concept of functional gradient materials was first proposed by Japanese researchers in 1978 and later expanded from aerospace engineering to building materials [10]. Craveiro et al. [11] created 3D-printed concrete with gradient thermal insulation properties by controlling cork filler distribution. However, their system mainly supports two-material mixing and does not enable sequential deposition of multiple independent slurries with layer-by-layer compositional control. Du Liping et al. [12] enhanced structural deformation resistance of building panels through dual-layer gradient structures combining ultra-high-performance concrete and lightweight aggregate concrete. Alarrak et al. [13] achieved localized toughening modification of cement-based composites through in-situ fiber injection technology. Despite these advancements, specialized printing equipment tailored for gradient material fabrication remains lacking. Regarding 3D printer nozzle optimization, existing designs fail to meet the technical requirements for multi-component gradient printing. The oblique rectangular nozzle developed by Nematollahi et al. [14] is only suitable for single-paste delivery scenarios, while Mechtcherine et al. [15] proposed an external synchronous filament deposition system that only enables external fiber toughening without achieving gradient control of matrix components. Overall, current nozzle optimization research primarily focuses on single-material delivery applications, and systematic parameter optimization for multi-cavity nozzles designed for layered multi-component extrusion remains unexplored. Recent studies on the rheological evaluation of printable cementitious slurries have highlighted the importance of yield stress and shear-thinning behavior in extrusion-based printing [16,17]. These studies provide a basis for the rheological characterization and numerical modeling adopted in this work.

To address the aforementioned technical bottlenecks, this study proposes innovative solutions across three dimensions: equipment structural design, parameter optimization, and integrated material–machine coordination. A unified printing nozzle was developed, featuring four independent auxiliary material chambers connected to a central main chamber. Equipped with servo-controlled material baffles and blade-type spiral structures, this nozzle enables sealed, independent delivery of various slurry types, designed to minimize interlayer mixing during gradient printing by isolating each slurry in an independently driven chamber with a servo-controlled baffle. The study here is limited to the structural measure adopted to suppress mixing, the operability of which is confirmed by the continuous printing tests reported in Section 3.2. Through computational fluid dynamics simulations, the study systematically elucidated how key structural parameters including auxiliary chamber pitch, main chamber pitch, and spindle rotation speed influence internal pressure distribution and outlet velocity, providing a preliminary basis for parameter selection under the adopted numerical assumptions. Utilizing this optimized gradient printing nozzle, multi-component structured gradient functional foam concrete was successfully fabricated. The graded concrete showed improved mechanical strength and resistance to repeated impacts relative to the homogeneous control. However, its thermal insulation performance and suitability for island infrastructure require further experimental evaluation.

## 2. Structural Design and Testing Methods of Printing Nozzles

### 2.1. Structural Design of Functionally Hierarchical Printing Nozzles

**Figure 1:**
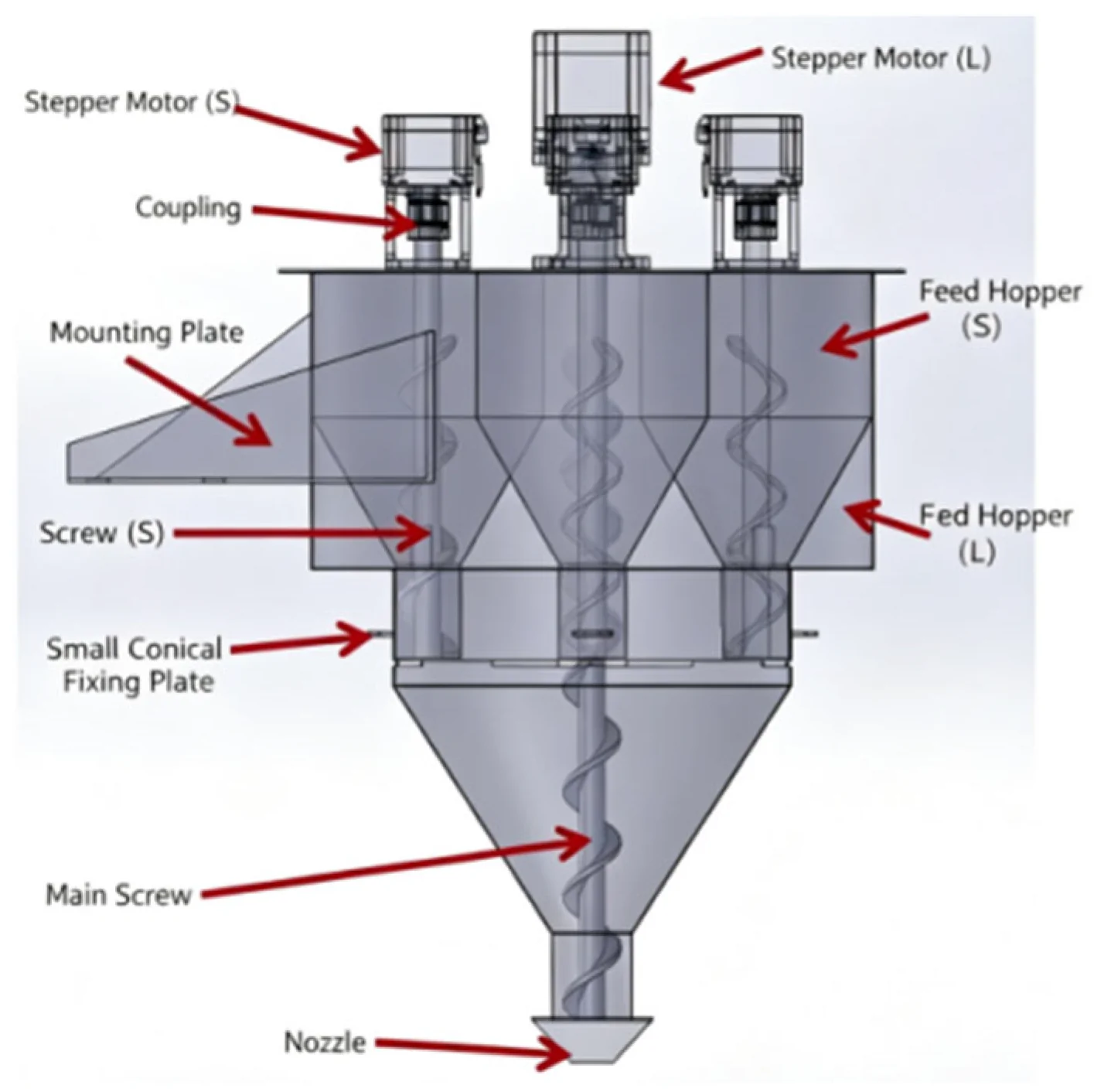
Schematic diagram of the printing nozzle structure.

As shown in Figure 1, the nozzle consists of four independent small material chambers, a central main material cylinder, an 86-step stepper motor drive system (with a rated torque of 8.5 N·m), a DS3225 servo-controlled cutting baffle (Dongguan Dsservo Technology Co., Ltd., located in Dongguan City, Guangdong Province, China), a blade-type screw conveying mechanism, and a conical discharge port; the material cylinder has an inner diameter of 50 mm and a height of 110 mm. Each material chamber is independently driven by a screw, and its bottom servo baffle operates separately to prevent slurry mixing during layered feeding; instead of using a full-blade screw, a blade structure was adopted, which is suitable for foam-based viscous plastic slurries, enabling synchronous conveying and stirring to prevent fiber agglomeration; the conical nozzle optimizes discharge uniformity and reduces flow dead zones.

### 2.2. Cfd Simulation Settings

#### 2.2.1. Model and Grid Division

**Figure 2:**
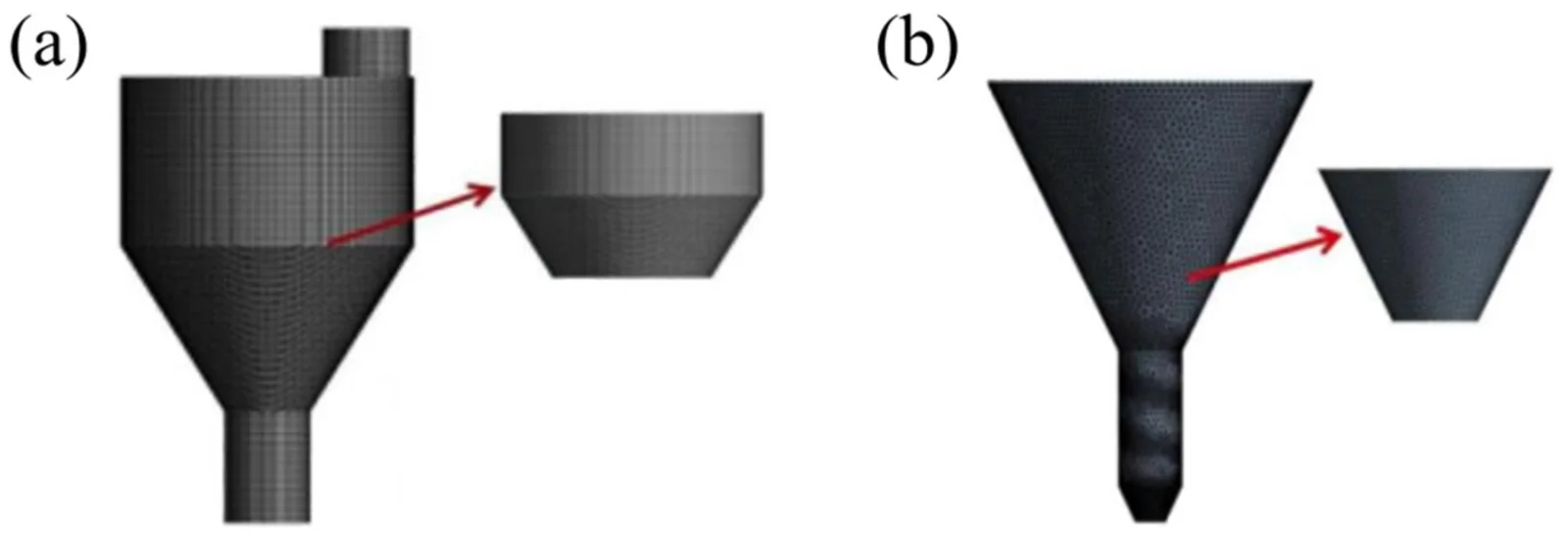
Finite element meshing diagram: (**a**) grid division of the small storage bin; (**b**) grid division of the large storage bin.

A 3D model was created using SolidWorks 2023 and imported into Design Modeler module of ANSYS Workbench 2024 R2, with minor fillets removed to simplify the geometry. The simulation model employed tetrahedral meshing, with localized refinement at the screw and nozzle outlet (element size: 2 mm). Two meshes containing approximately 400,000 and 800,000 elements were compared. The supporting test at an inlet velocity of 0.02 m/s and screw speed of 60 r/min reported maximum fluid velocities of 1.330 and 1.310 m/s, respectively, after 4 s, corresponding to a relative difference of approximately 1.5%. The 800,000-element mesh was adopted for the subsequent calculations (Figure 2). This two-mesh comparison evaluates the sensitivity of the maximum velocity in that test.

#### 2.2.2. Boundaries and Computational Conditions

The slurry has a density of 2300 kg/m^3^ (measured wet density of the fresh foam concrete) and an apparent dynamic viscosity of 13.83 Pa·s at a shear rate of 50 s^−1^, measured using an LBY-III rheometer. The rheological characterization showed shear-thinning behavior with a static yield stress of approximately 150 Pa. Within the shear rate range relevant to screw extrusion (10–100 s^−1^), the apparent viscosity variation was moderate, and an equivalent Newtonian viscosity was adopted as a first-order approximation. The Reynolds number calculated based on the inlet velocity (0.03 m/s), characteristic length (50 mm), density, and apparent viscosity is approximately 0.25, indicating laminar-dominated flow. The standard k-ε turbulence model was employed for numerical stability, acknowledging that the actual flow regime is laminar. The inlet velocity at the top is 0.03 m/s, and the outlet pressure is atmospheric. Gravitational acceleration of −9.8 m/s^2^ along the *Y*-axis was applied, and continuity and momentum conservation equations were included.

### 2.3. Raw Materials and Reference Mix Ratios

This experiment employed RSAC42.5 sulfoaluminate cement as the matrix cementitious material, with an initial setting time of 10 min and a final setting time of 15 min; its primary chemical components are CaO and SiO_2_. The experimental mixtures included silica fume (specific surface area: 18.64 m^2^/g; SiO_2_ content: 90.02%), Class I fly ash, natural river sand, and basalt fibers with a single fiber diameter of 0.2 mm and a length gradient of 6 mm. The admixture formulation consisted of a polycarboxylate superplasticizer, hydroxypropyl methylcellulose (HPMC; viscosity: 200 Pa·s) as an anti-dispersant, and a foaming agent. The mixing water used complied with municipal tap water standards specified for concrete applications. The chemical composition and functional gradation ratios for the silica fume mixture are detailed in Table 1 and Table 2, where O1 represents a formulation containing 1% basalt fibers, O2 denotes a high sand-to-cement ratio formulation, and O3 indicates a formulation with 40% foaming agent. The foaming agent (protein-based) was diluted to 5% concentration, pre-foamed by high-speed stirring (3000 rpm, 3 min) to a foam density of 80 ± 5 g/L, and then incorporated into the slurry by volume. The measured dry densities of O1, O2, and O3 were approximately 1030, 1180, and 850 kg/m^3^, respectively. Printing parameters were as follows: layer height 10 mm, printing speed 40 mm/s, interlayer interval about 30 s, and curing at 20 ± 2 °C and RH ≥ 95%.

### 2.4. Printing Process and Mechanical Property Testing

#### 2.4.1. Preparation of Graded Test Samples

**Figure 3:**
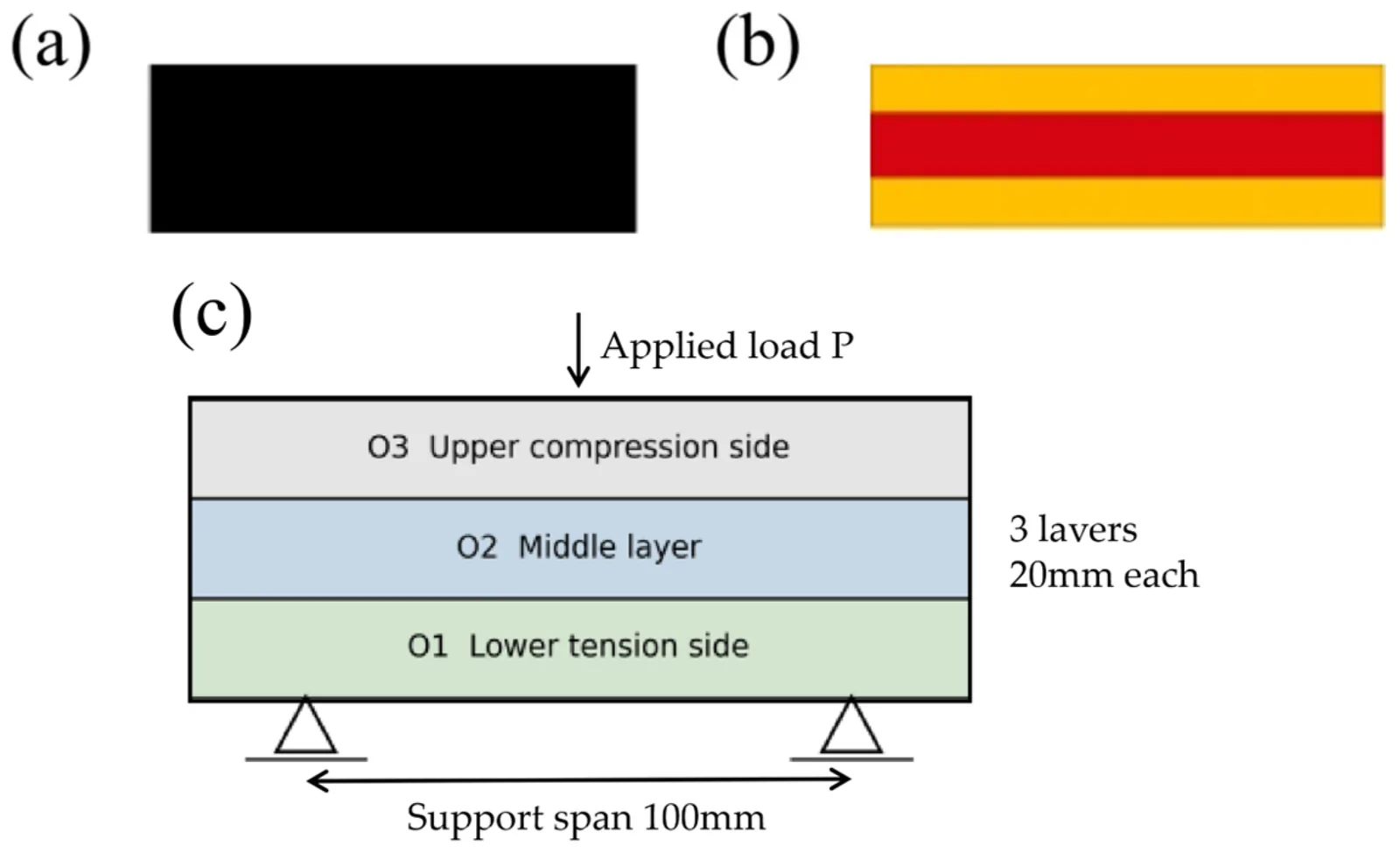
Diagram of the basalt fiber gradient foam concrete structure: (**a**) schematic diagram of a homogeneous monolayer structure; (**b**) schematic diagram of the three-layer structure. (**c**) H2 flexural-test orientation: H2 under central downward loading with two lower supports, showing O3 on the upper compression side, O2 in the middle, and O1 on the lower tension side. Panel (**c**) is schematic and not to scale.

The structural schematic is shown in Figure 3, employing a three-tiered layer combination design with each layer printed twice consecutively, resulting in a total of six printing layers for each specimen group [18]. Six layer sequence combinations were established, as listed in Table 3. Group H1 serves as the homogeneous control group (six consecutive layers of O3), while Groups H2 to H6 represent functionally graded specimens with different layer sequences but identical constituent materials, so that the effect of layer sequencing can be isolated by comparing H2–H6. The specimen dimensions are as follows: flexural specimens measure 200 mm × 40 mm × 60 mm, and compressive specimens measure 40 mm × 40 mm × 60 mm; each functional layer corresponds to two consecutive deposition passes with a nominal layer height of 10 mm, and the layers were deposited in immediate succession to ensure fresh-on-fresh interlayer bonding. Three specimens were tested per group at each curing age, and the reported strength values are the means ± standard deviations. The flexural test used a support span of 100 mm and a loading rate of 0.5 mm/min; the compressive test used a loading rate of 1.0 mm/min, in accordance with GB/T 17671-2021. All specimens were subsequently placed in the SHBY-40B constant temperature and humidity curing chamber (from Hebei Xinmeihua Testing Instrument Co., Ltd., located in Cangzhou City, Hebei Province, China) for standard curing, with mechanical property characterization conducted at curing ages of 1 day, 7 days, and 28 days.

#### 2.4.2. Testing Standards and Equipment

The compressive and flexural tests of the specimens were conducted using the CMT-5305 electronic universal testing machine (MTS Systems (China) Co., Ltd., Shenzhen, China) in accordance with the GB/T 17671-2021 standard [19]. The drop hammer impact test was performed under ACI 544 specifications [20], employing a 4.5 kg steel ball dropped from a height of 600 mm, with initial crack load and macroscopic failure morphology recorded. Extrusion performance was quantitatively evaluated based on extruded slurry mass per minute (g/min).

## 3. Results and Discussion

### 3.1. CFD Analysis of the Internal Flow Field in the Nozzle

#### 3.1.1. Effect of Screw Pitch of the Feed Hopper on Pressure and Velocity

**Figure 4:**
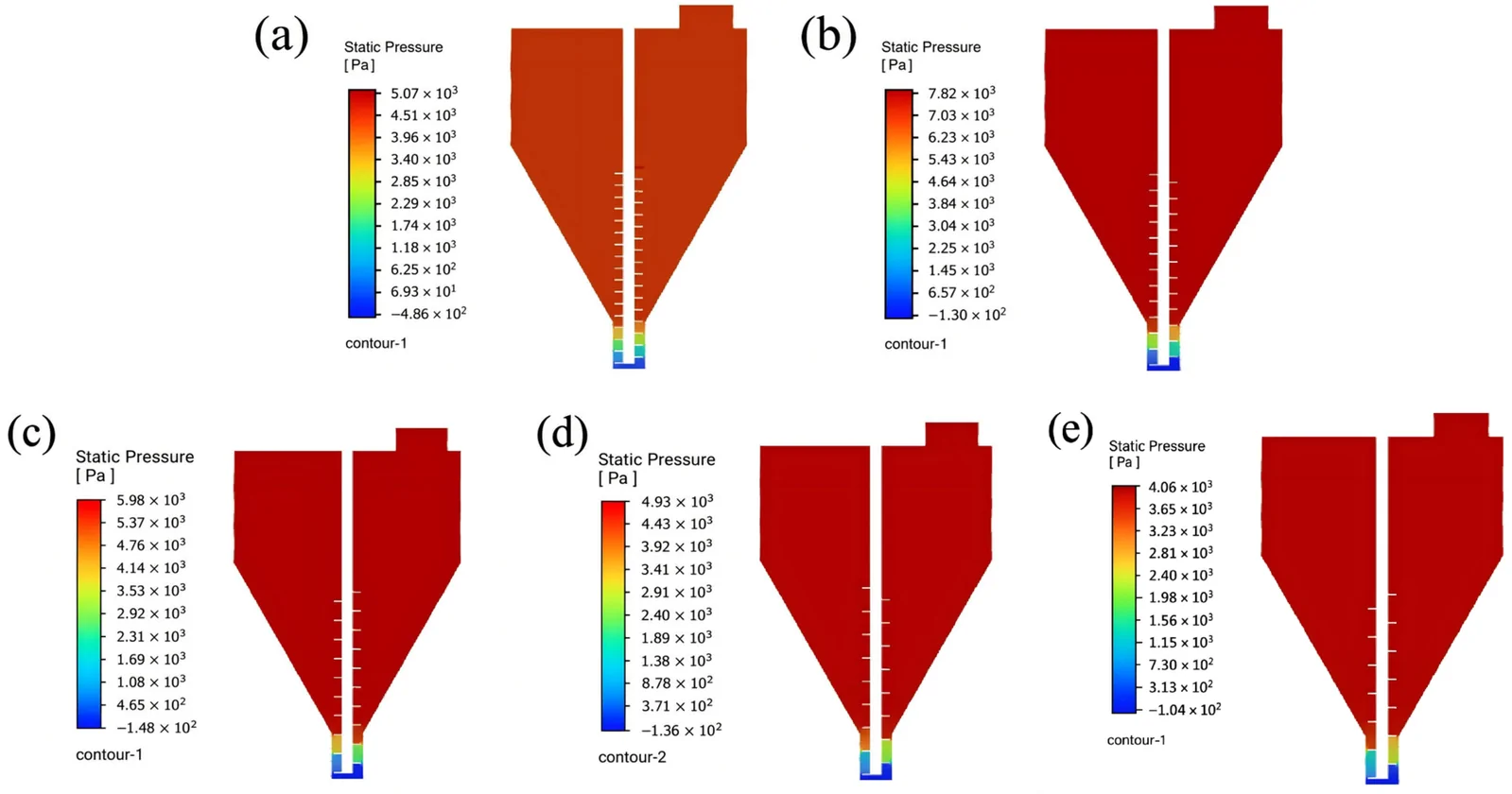
Pressure contour maps of the fluid region in the small silo at different pitch values: (**a**) 7.5 mm pitch; (**b**) 10 mm pitch; (**c**) 12.5 mm pitch; (**d**) 15 mm pitch; (**e**) 17.5 mm pitch.

**Figure 5:**
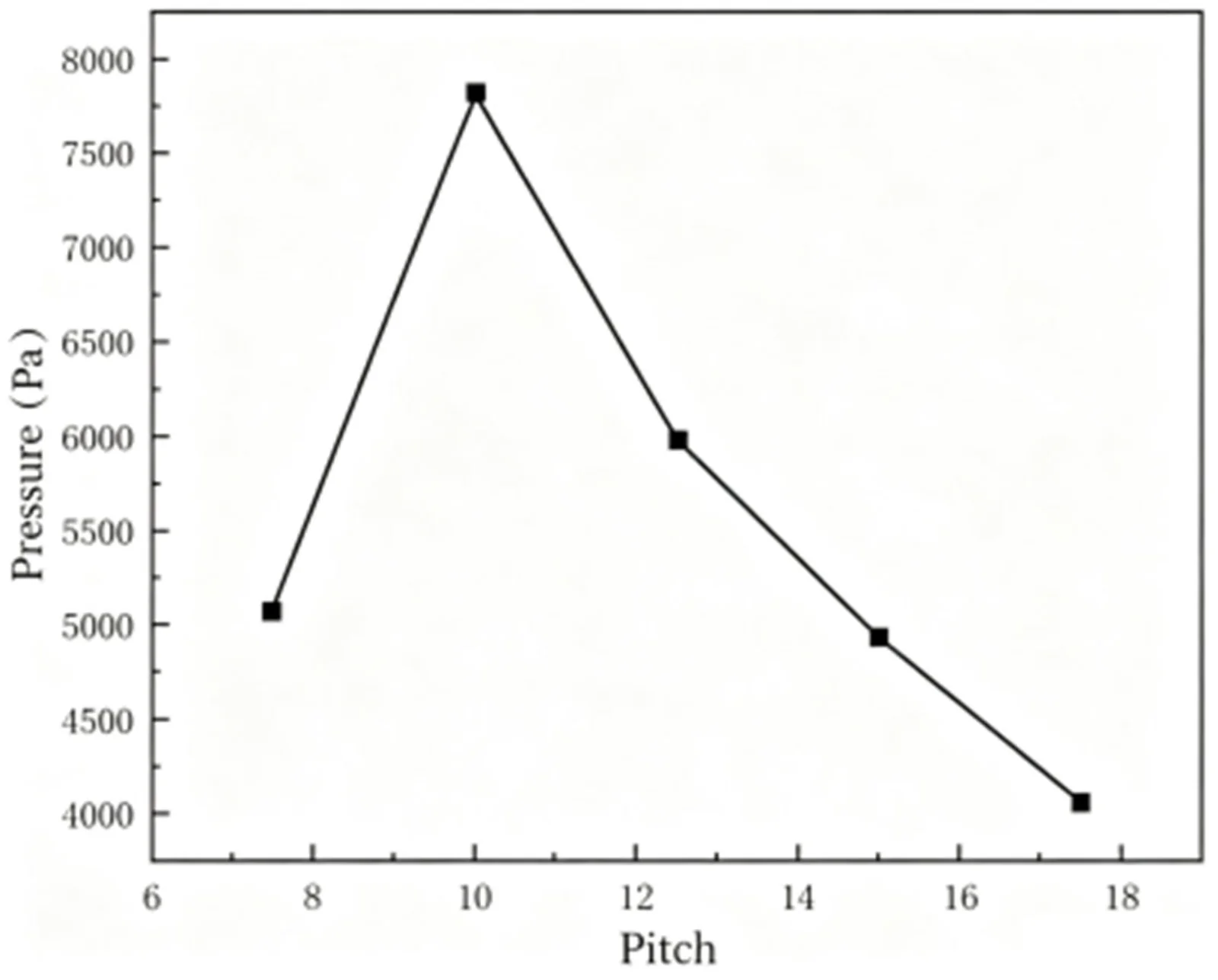
Pressure variation graph.

The simulation results are shown in Figure 4; as illustrated in Figure 5, the internal static pressure within the small material chamber exhibits a unimodal variation trend that first increases and then decreases with increasing screw pitch. When the screw pitch increases from 7.5 mm to 10 mm, the chamber pressure rises rapidly from 5070 Pa to a peak of 7820 Pa, attributed to intensified extrusion, shearing, and compaction effects caused by reduced spiral groove space. Beyond 10 mm screw pitch, the expansion of the helical channel enhances material transport rate and shortens retention time, thereby weakening accumulation and compression effects [21], with chamber pressure gradually declining to 4060 Pa. The 10 mm screw pitch marks the critical peak pressure point where slurry flow resistance reaches its maximum; using a greater screw pitch effectively reduces chamber pressure loads, optimizes slurry flow efficiency, and ensures continuous and stable extrusion during printing.

**Figure 6:**
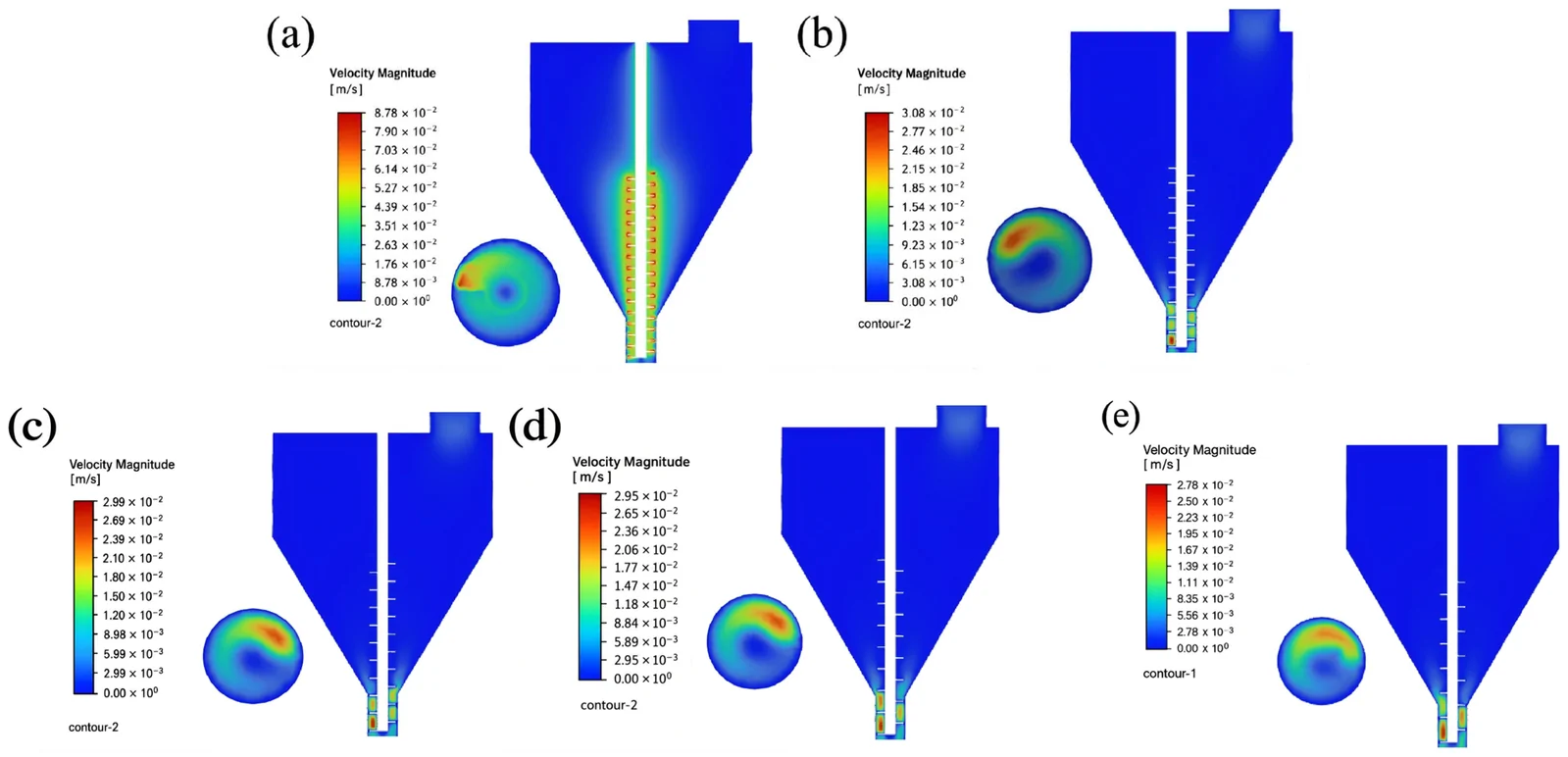
Velocity contour map of the fluid region in the small silo at different pitch values: (**a**) 7.5 mm pitch; (**b**) 10 mm pitch; (**c**) 12.5 mm pitch; (**d**) 15 mm pitch; (**e**) 17.5 mm pitch.

**Figure 7:**
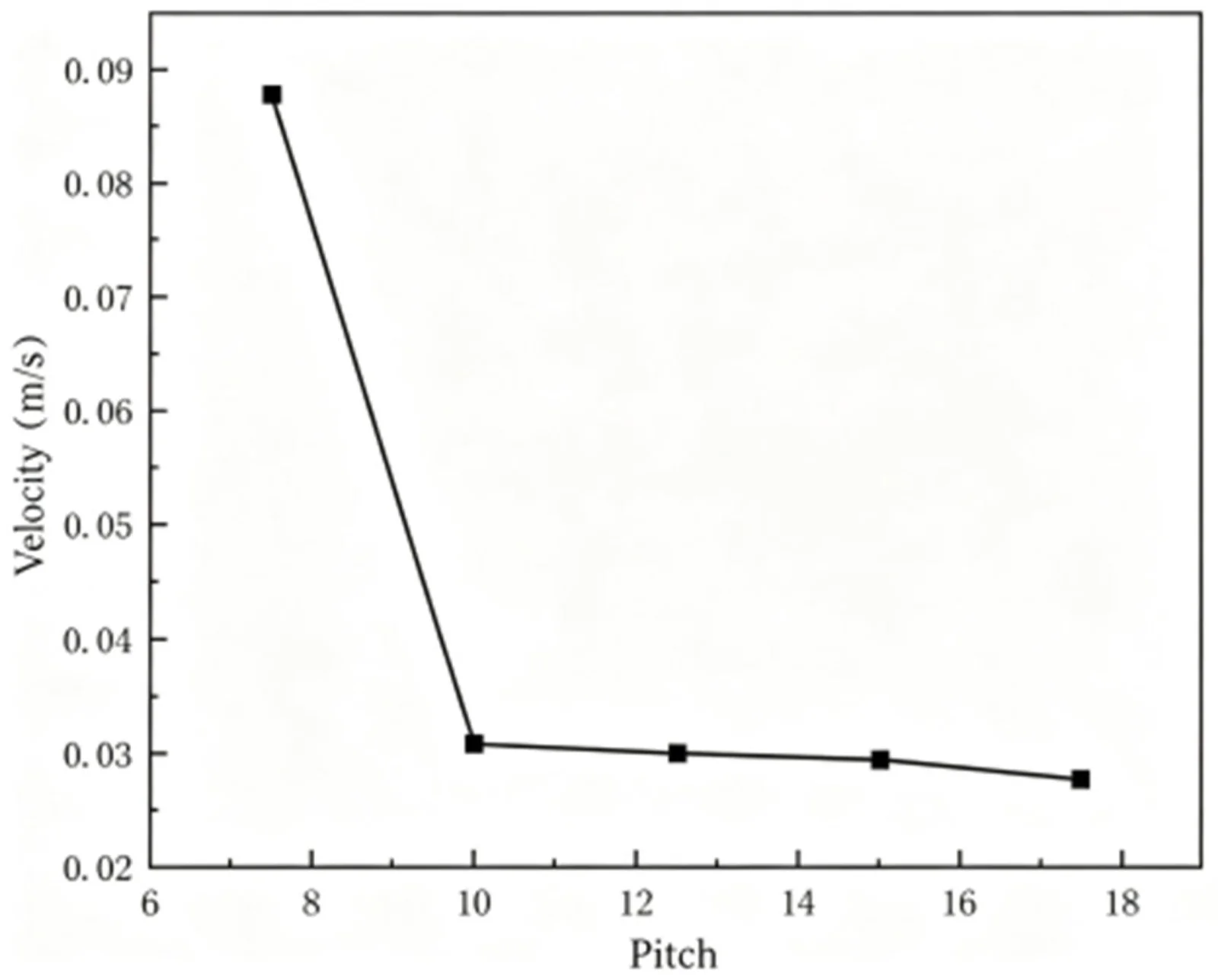
Velocity profile.

**Figure 8:**
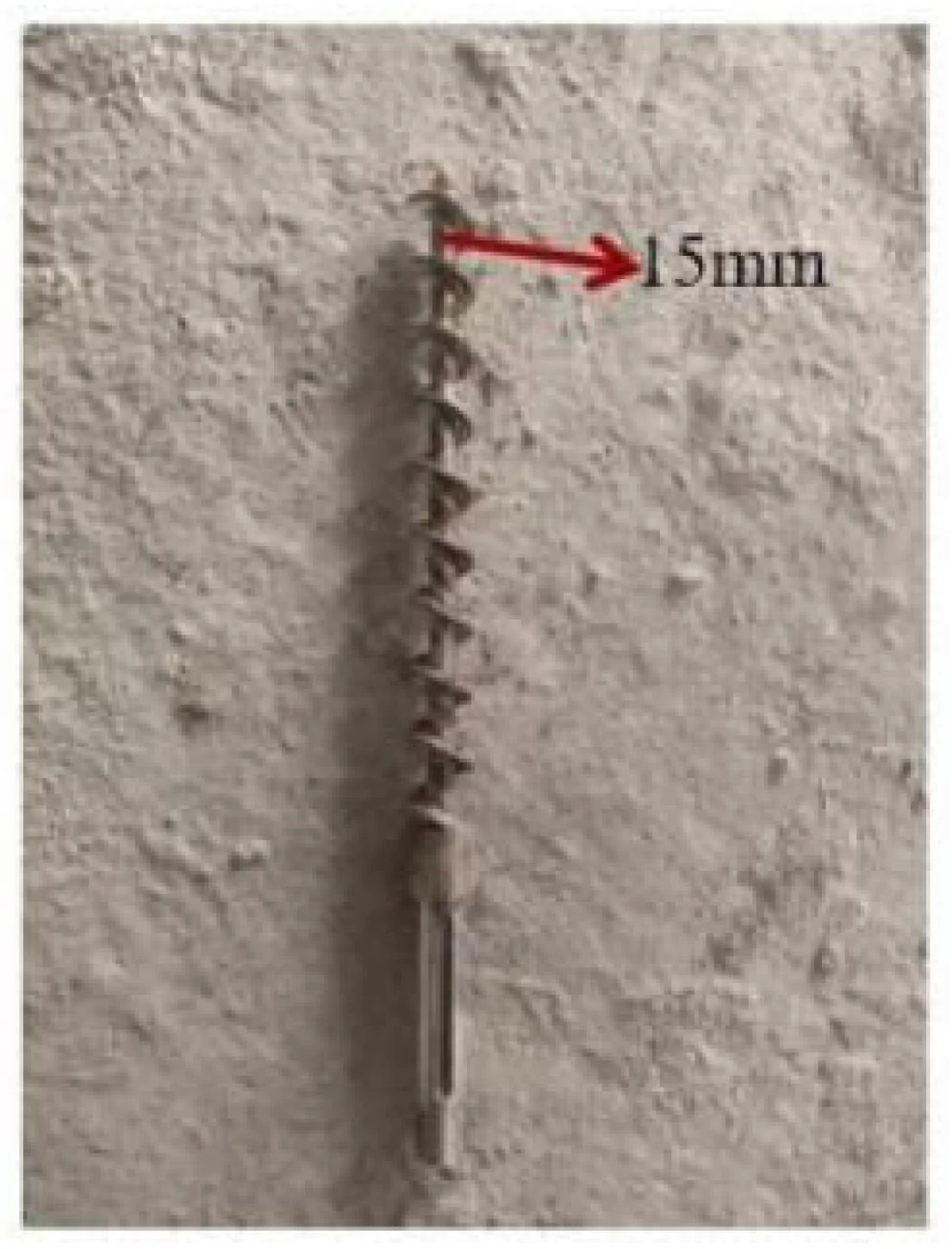
Detail drawing of the auxiliary screw.

The simulation results are illustrated in Figure 6. As shown in Figure 7, the discharge velocity from the small hopper exhibits a characteristic evolution pattern: a sharp decline followed by gradual stabilization as screw pitch increases. When the pitch rises from 7.5 mm to 10 mm, the discharge velocity drops abruptly from 80 mm/s to 30 mm/s. A smaller pitch corresponds to higher spiral chamber filling rates and greater instantaneous feeding volumes, while increased pitch slows material transportation efficiency. For pitches exceeding 10 mm, discharge velocity shows minor fluctuations and stabilizes within the range of 27–30 mm/s, with the regulatory effect of pitch expansion on flow velocity significantly diminishing. Comprehensive analysis of chamber pressure and velocity trends identifies 10 mm as the critical pitch threshold for flow behavior transition—where static chamber pressure peaks at 7820 Pa, potentially causing localized material compression and clogging issues that hinder uniform extrusion. While pitches below 10 mm achieve higher discharge rates, they introduce high chamber pressure and flow instability. Pitches above 10 mm maintain stable chamber pressure and consistent discharge rates, ensuring more reliable and controllable slurry transport. Considering key requirements such as low chamber pressure, stable discharge speed, and transportation efficiency, 15 mm is selected as the optimal pitch for the small hopper. The physical prototype of the auxiliary screw is depicted in Figure 8.

#### 3.1.2. Effect of Pitch on Pressure and Velocity in the Main Material Chamber

**Figure 9:**
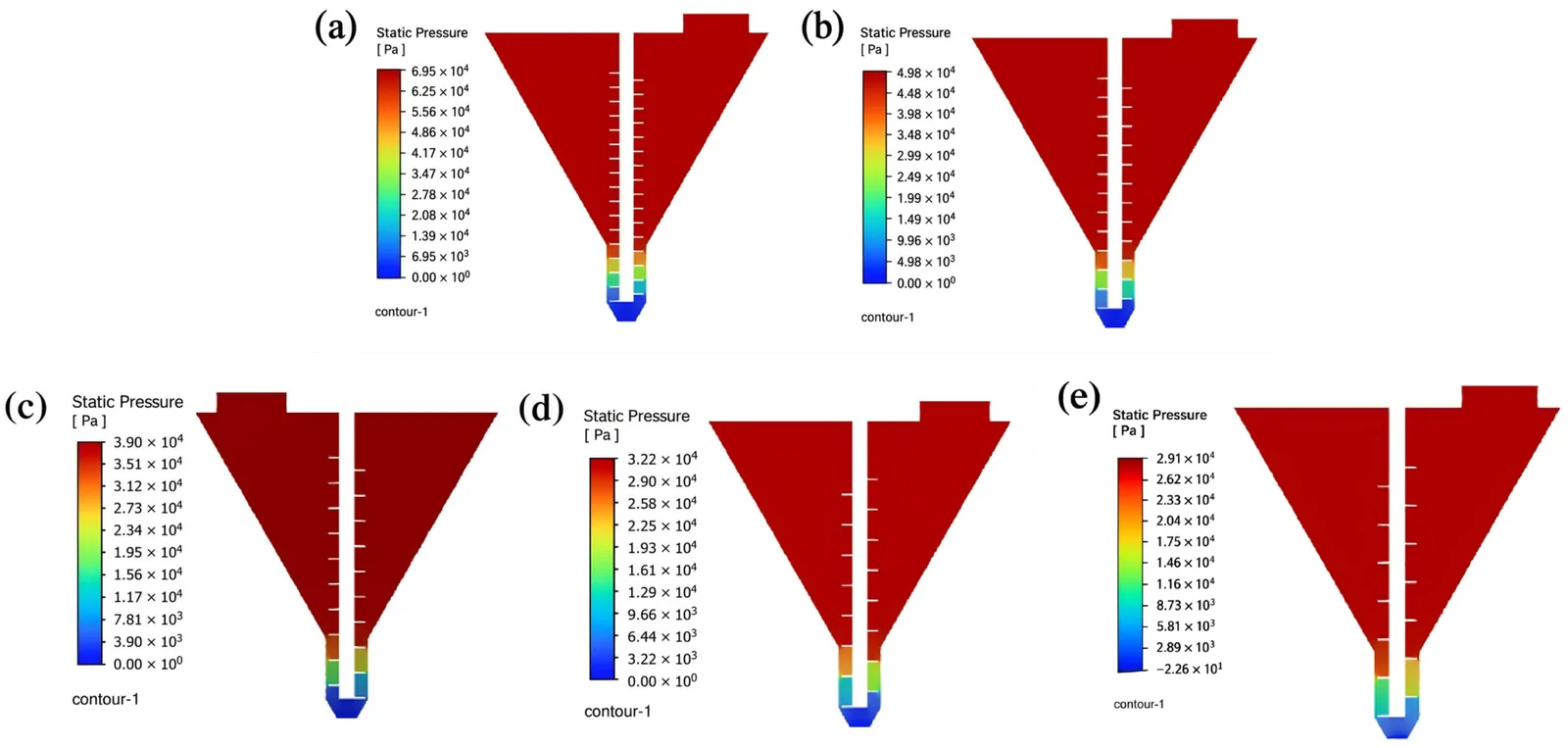
Pressure contour map of the fluid area in the large silo at different pitch values: (**a**) 15 mm pitch; (**b**) 20 mm pitch; (**c**) 25 mm pitch; (**d**) 30 mm pitch; (**e**) 35 mm pitch.

**Figure 10:**
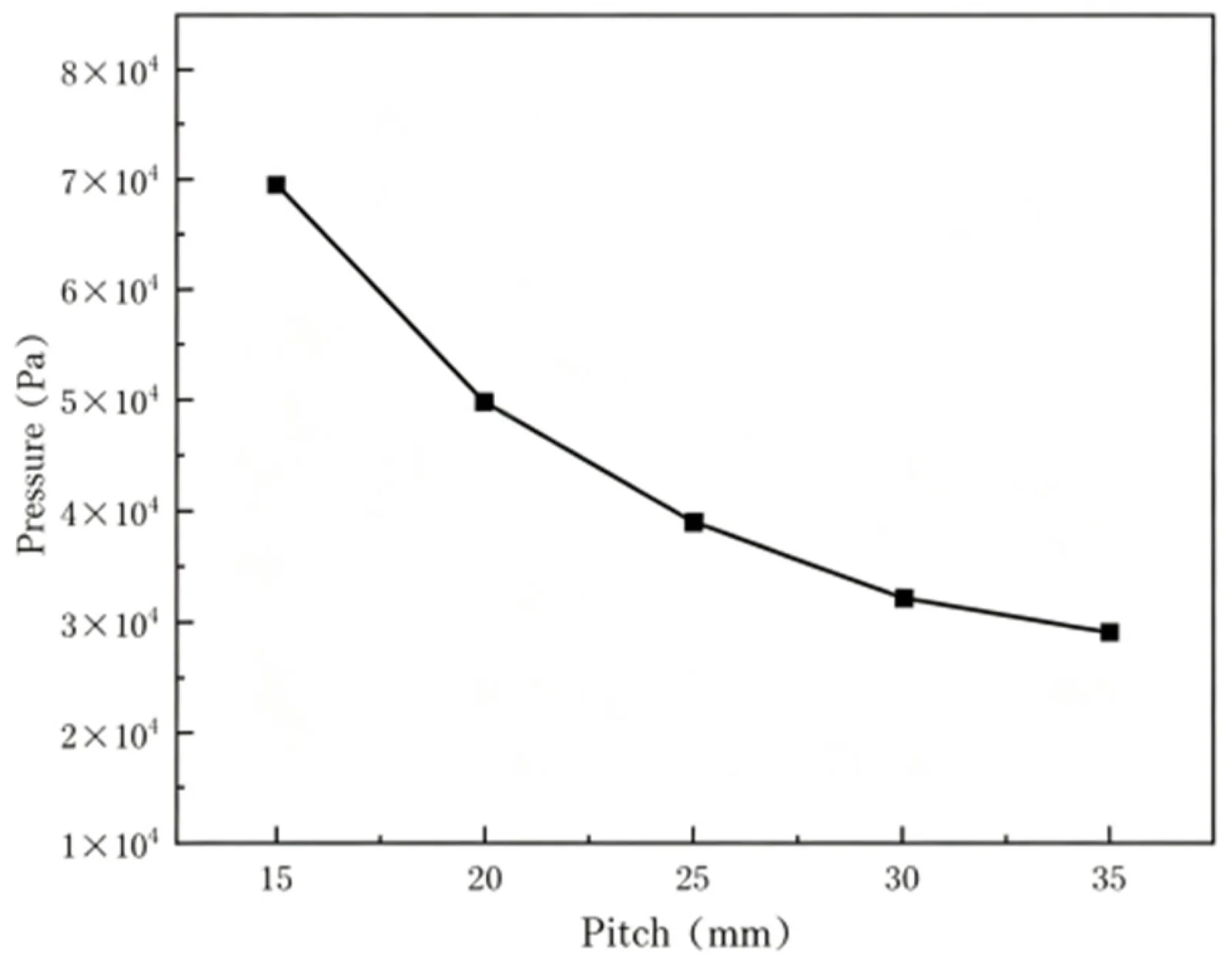
Pressure variation graph.

The simulation results are shown in Figure 9. As illustrated in Figure 10, the static pressure inside the main hopper decreases monotonically with increasing screw pitch. When the pitch varies between 15 and 35 mm, the chamber pressure drops steadily from 7 × 10^4^ Pa to 3 × 10^4^ Pa without any significant abrupt changes. The increased pitch widens the spiral conveying channel, shortens the slurry retention time, and continuously reduces material accumulation and compression effects, thereby achieving a steady pressure decline within the chamber. This gradual variation pattern demonstrates that the adjustable pitch range of 15–35 mm enables continuous optimization of slurry stress distribution and flow dynamics, mitigates local stress concentrations and flow distortions, and provides a comprehensive parameter selection range for stable extrusion in the main hopper.

**Figure 11:**
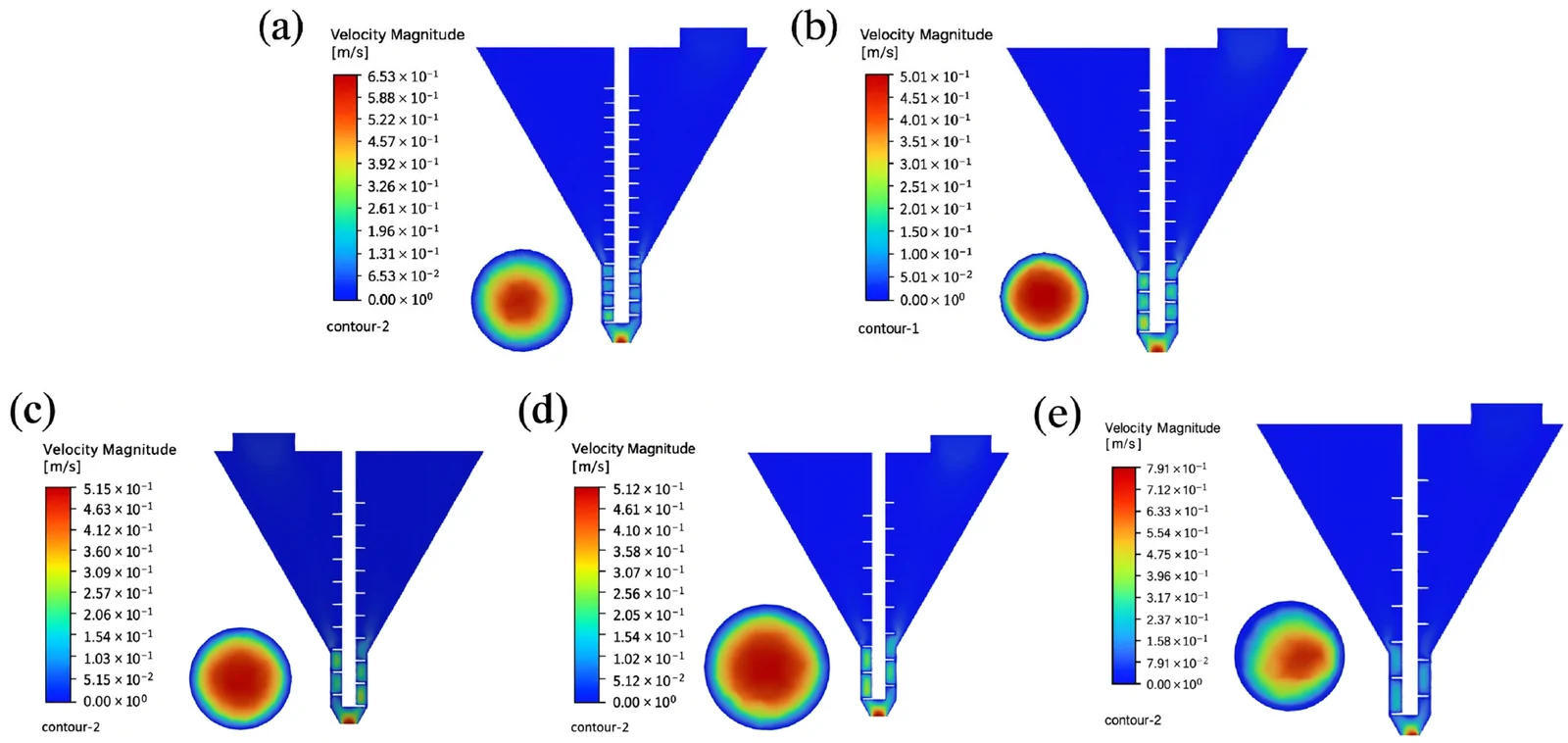
Velocity cloud diagrams of fluid regions in the large silo under different pitch conditions: (**a**) 15 mm pitch; (**b**) 20 mm pitch; (**c**) 25 mm pitch; (**d**) 30 mm pitch; (**e**) 35 mm pitch.

**Figure 12:**
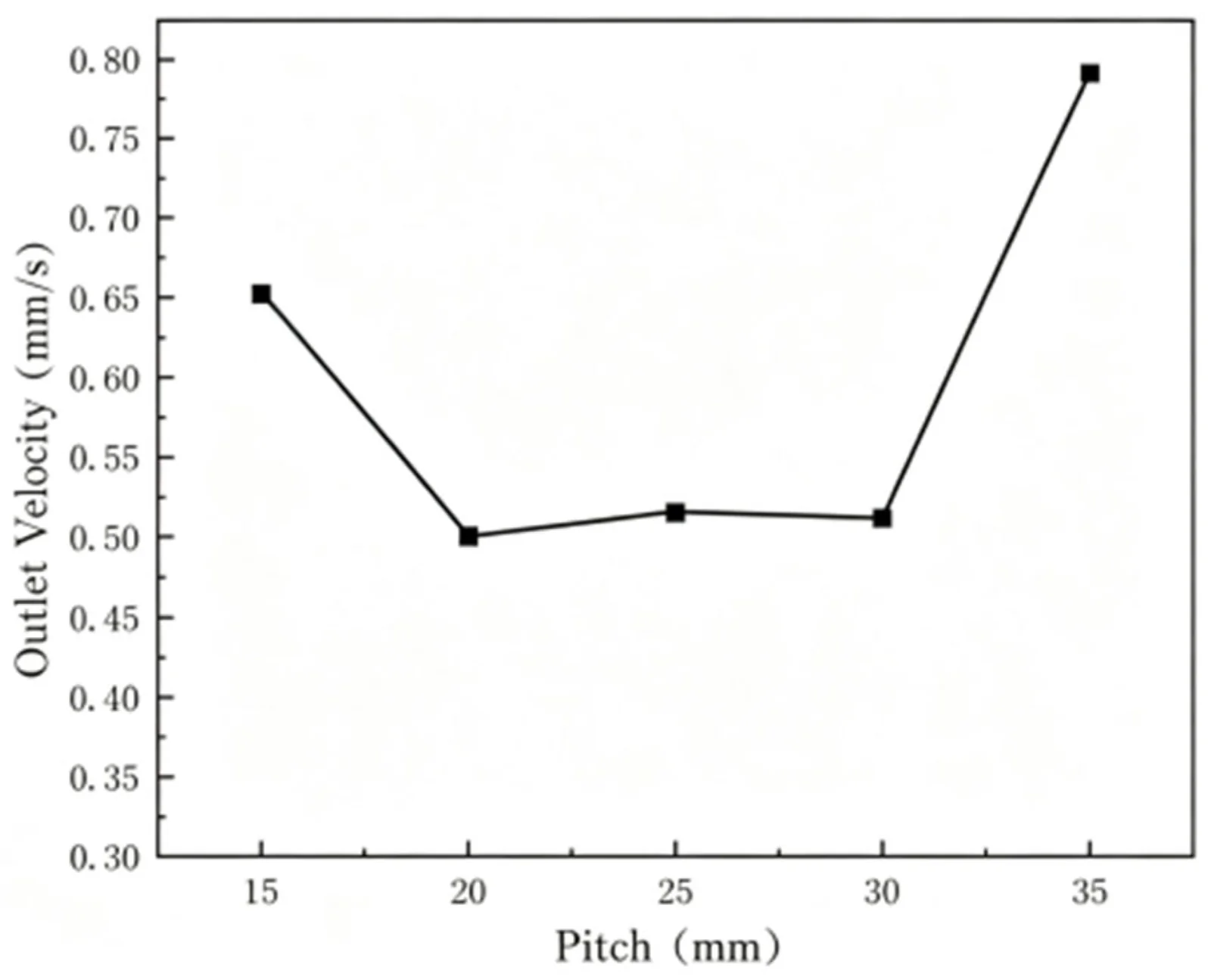
Velocity profile.

The simulation results are shown in Figure 11; as illustrated in Figure 12, the discharge velocity from the large hopper exhibits a pattern that first decreases, then stabilizes, and finally increases sharply with increasing pitch: when the pitch increases from 15 mm to 20 mm, the outlet velocity declines from 0.65 m/s to 0.50 m/s, indicating a slowdown in material feeding speed; within the range of 20–30 mm, the flow velocity remains stable at 0.50–0.52 m/s, ensuring steady slurry conveyance; when the pitch reaches 35 mm, the outlet velocity rises abruptly to 0.79 m/s. In conclusion, the range of 20–30 mm represents the optimal pitch interval for stable feeding in the large hopper; although the 35 mm configuration offers higher discharge efficiency, its abrupt flow velocity change may lead to uneven flow fields, necessitating comprehensive evaluation based on chamber pressure to determine its engineering applicability.

**Figure 13:**
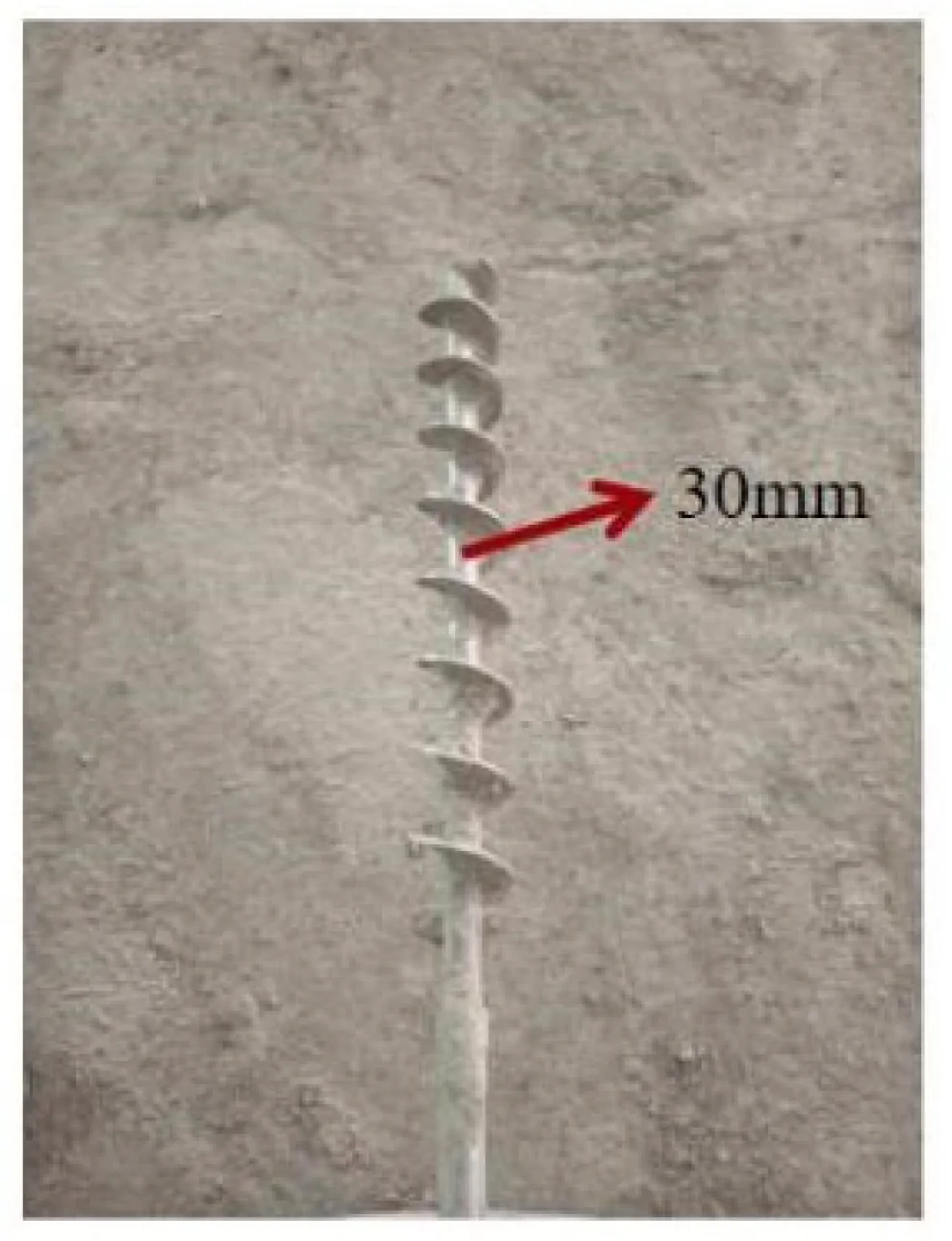
Main screw physical drawing.

Based on the analysis of the evolution patterns of static pressure and discharge outlet velocity in the large material chamber, within a pitch range of 15–35 mm, chamber pressure decreases monotonically from 7 × 10^4^ Pa to 3 × 10^4^ Pa, while the outlet velocity exhibits a pattern of initial decline, stabilization, followed by a sharp increase: from 15 to 20 mm, the outlet velocity drops from 0.65 m/s to 0.50 m/s; from 20 to 30 mm, it stabilizes at 0.50–0.52 m/s; when the pitch increases to 35 mm, the outlet velocity rises sharply to 0.79 m/s. The range of 20–30 mm is optimal for the main material chamber, as it ensures steady pressure reduction, minimizes slurry compression and equipment load, and keeps a constant outlet velocity—conditions which are ideal for achieving uniform extrusion in 3D printing. A sudden increase in outlet velocity at 35 mm could lead to material dispersion; therefore, the optimal pitch for the main screw is determined to be 30 mm, as shown in Figure 13.

#### 3.1.3. Effect of Silo Rotation Speed on Pressure and Velocity

**Figure 14:**
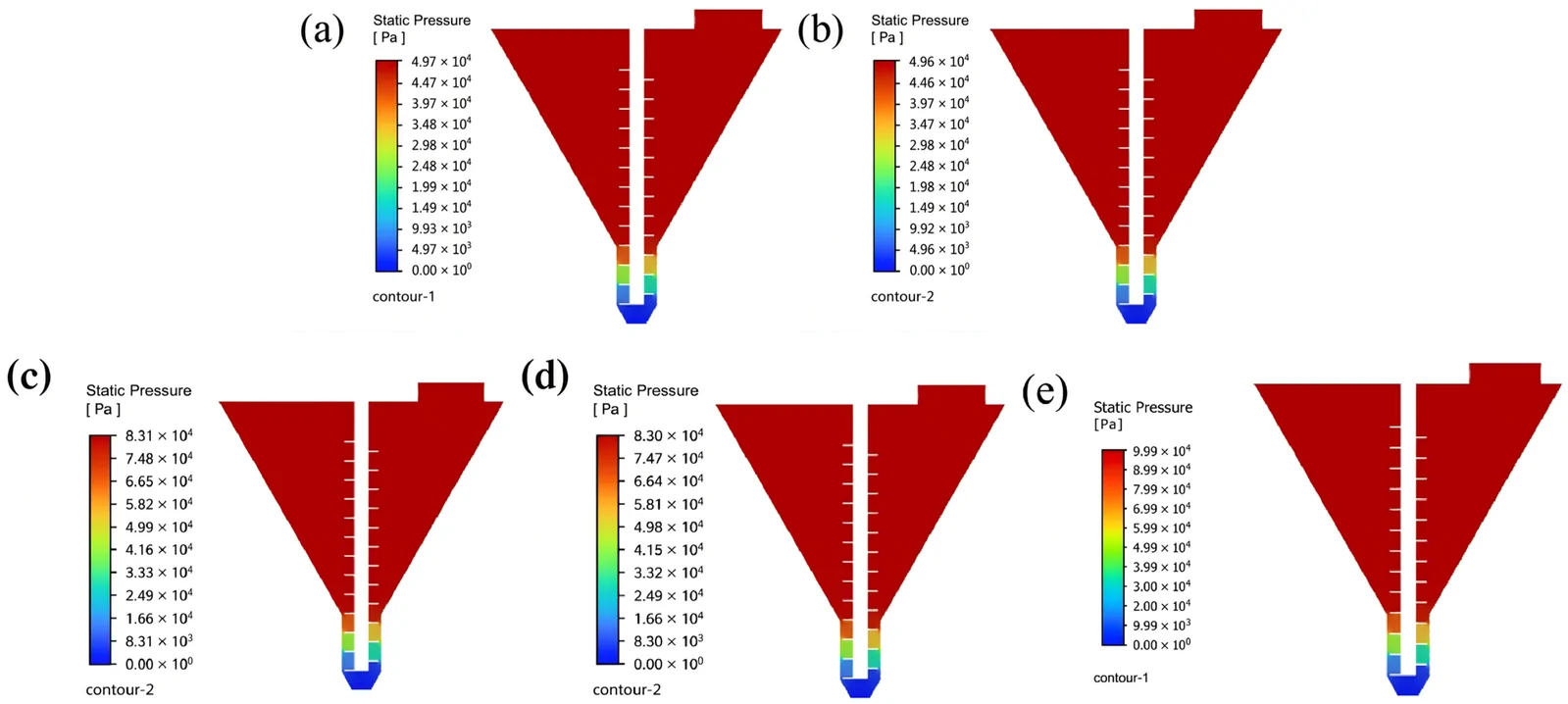
Pressure contour maps of the fluid area in the large silo under different screw rotation speeds: (**a**) 20 r/min; (**b**) 30 r/min; (**c**) 40 r/min; (**d**) 50 r/min; (**e**) 60 r/min.

**Figure 15:**
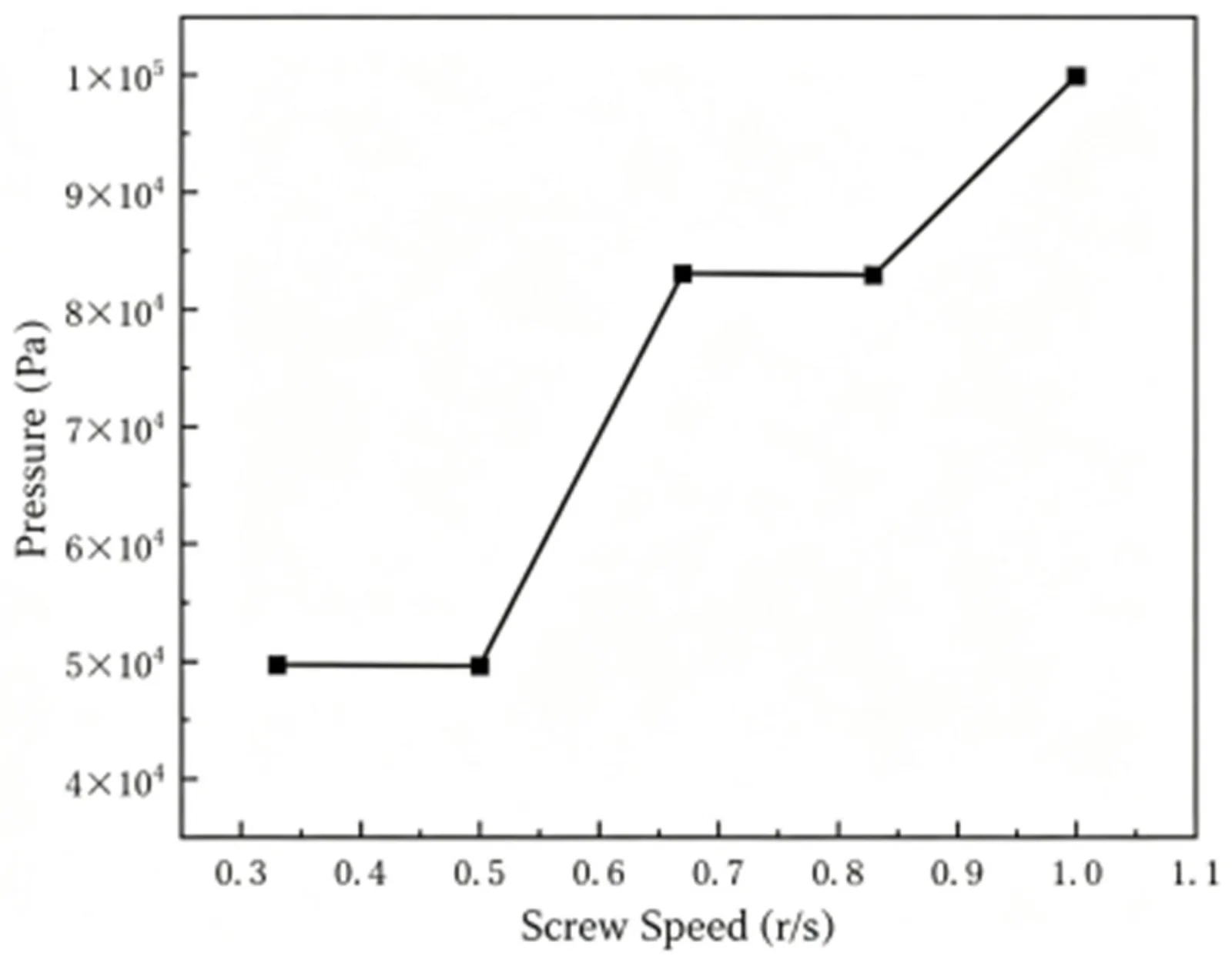
Pressure variation graph.

The simulation results are shown in Figure 14; as illustrated in Figure 15, the static pressure in the large material chamber increases stepwise with rising screw speed: at speeds of 20–30 r/min, the chamber pressure remains stable within the low-load range of 5 × 10^4^ Pa; when the speed rises to 40 r/min, the pressure rapidly increases to 8.3 × 10^4^ Pa and stabilizes between 40 and 50 r/min; further increasing the speed to 60 r/min raises the pressure again to 1 × 10^5^ Pa. Under low-speed conditions, slurry discharge is smooth and internal chamber forces are balanced; as speed increases, screw shear and material compression effects intensify, causing segmented pressure elevations across the chamber, with each corresponding to a distinct phase of flow equilibrium for the slurry.

**Figure 16:**
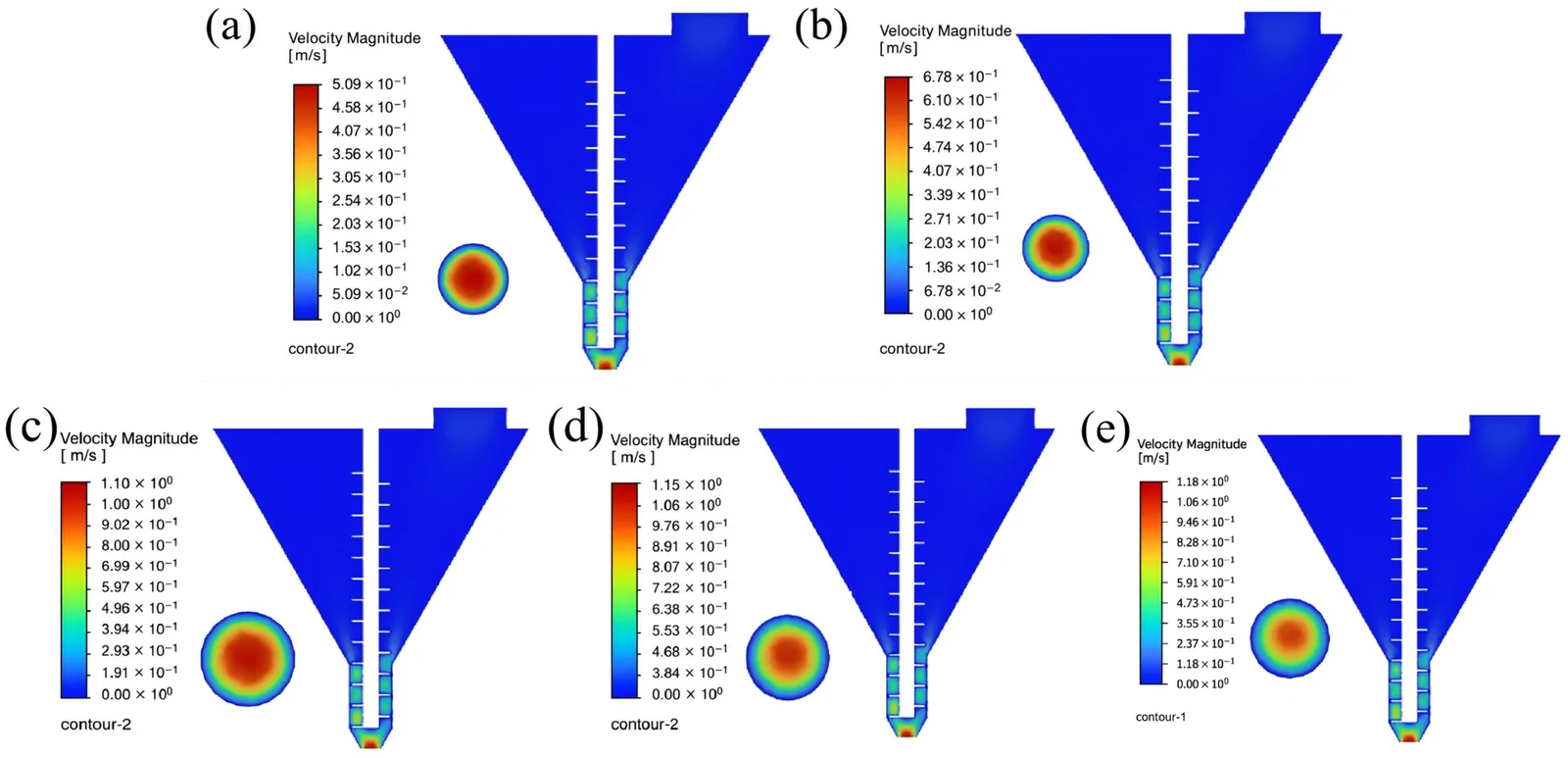
Velocity cloud diagrams of the fluid area in the large silo under different screw rotation speeds: (**a**) 20 r/min; (**b**) 30 r/min; (**c**) 40 r/min; (**d**) 50 r/min; (**e**) 60 r/min.

**Figure 17:**
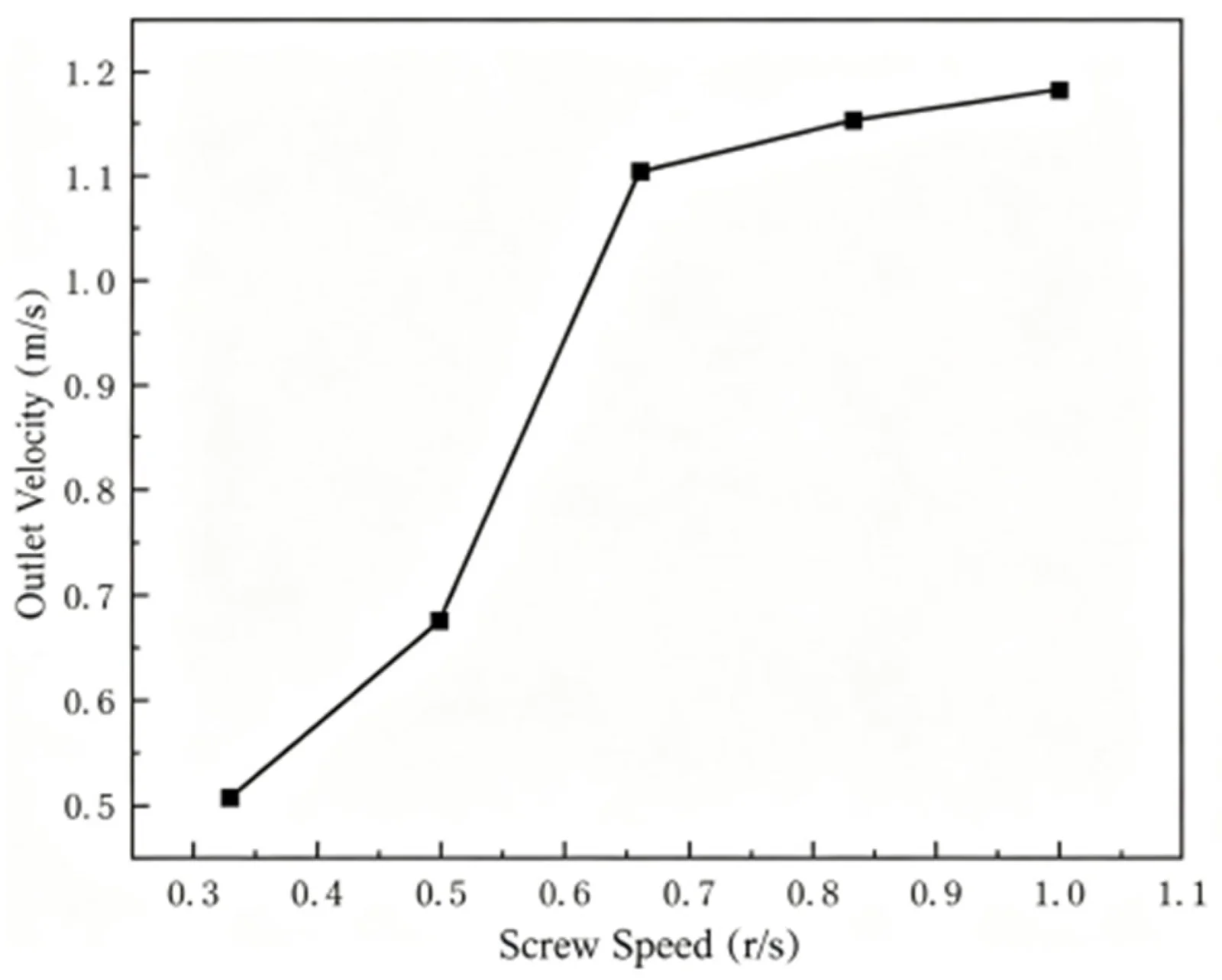
Velocity profile.

The simulation results are shown in Figure 16; as illustrated in Figure 17, the outlet velocity from the large hopper exhibits a trend of initially rising rapidly followed by a gradual slowdown as the screw speed increases. At speeds between 20 and 40 r/min, the outlet velocity rises sharply from 0.509 m/s to approximately 1.10 m/s. Beyond 40 r/min, the outlet velocity growth slows markedly, reaching approximately 1.15 m/s at 50 r/min and 1.18 m/s at 60 r/min, indicating saturation in slurry conveyance; further acceleration may lead to high chamber pressure and equipment overload. The 40 r/min speed represents the critical threshold of the outlet-velocity response, balancing delivery efficiency with extrusion stability.

By analyzing the coupling relationship between screw speed and both internal static pressure and the outlet velocity, the optimal operating speed for the large material chamber screw was determined to be 40 rpm. Under this condition, the chamber pressure remains stable at 8.3 × 10^4^ Pa with an outlet velocity of 1.10 m/s. This configuration ensures optimal printing efficiency while preventing slurry overpressure-induced compression and discharge pulsation, enabling continuous, homogeneous extrusion of the slurry and effectively guaranteeing the quality of 3D-printed components. The screw pitch and rotational speed were selected according to the following criteria: (i) a low chamber pressure to avoid material over-compression and nozzle clogging; (ii) a stable outlet velocity to ensure uniform extrusion; and (iii) an adequate throughput for continuous printing. These criteria were further validated experimentally by continuous printing and by the measured steady extrusion rate (Section 3.2).

### 3.2. Verification of Nozzle Printing Performance

**Figure 18:**
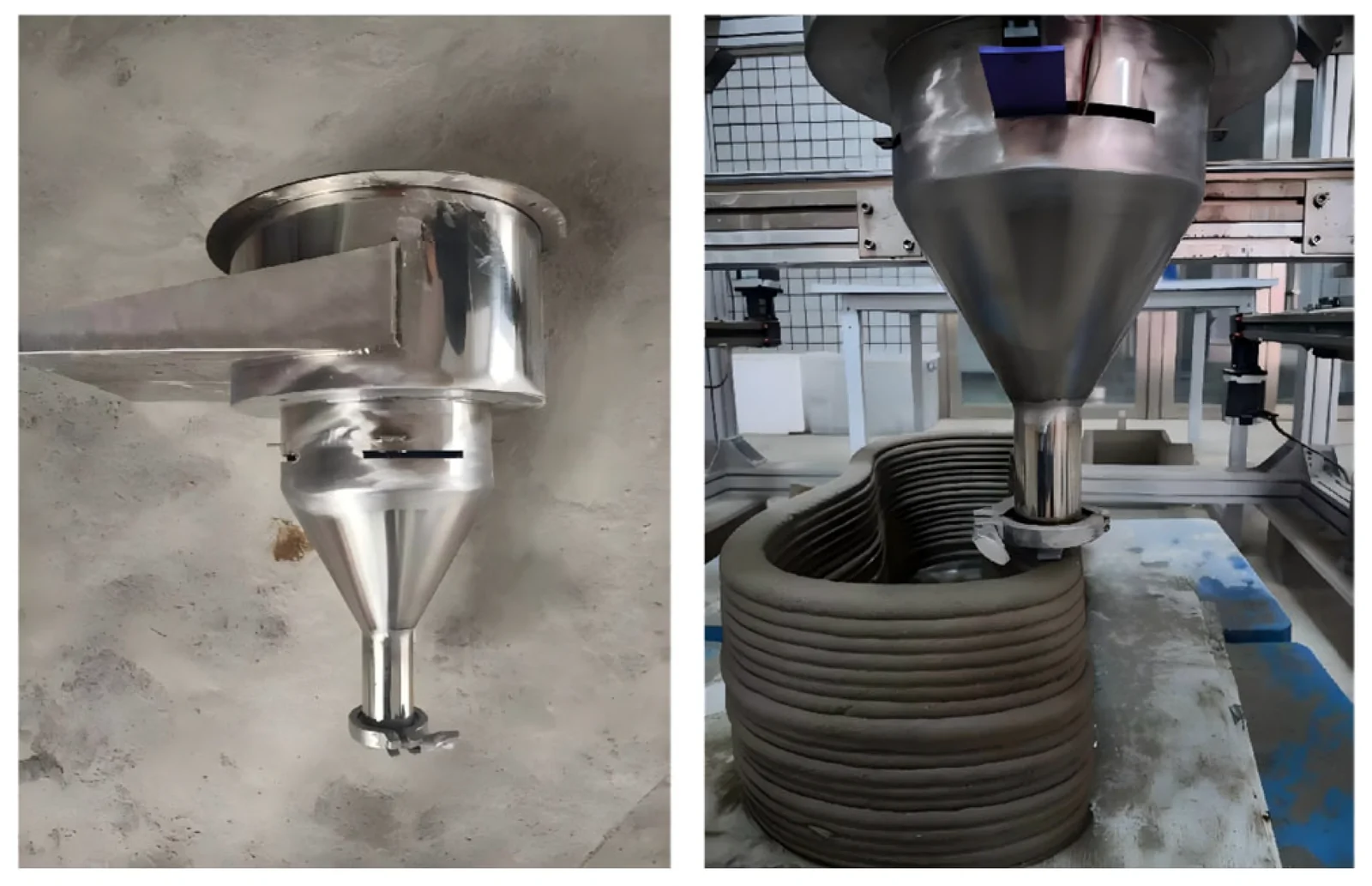
Printed sample.

Using the optimized parameter processing prototype, continuous printing was performed for 60 min without interruption, and the extrusion rate remained stable at 833 g/min (measured as the extruded mass per minute at 40 r/min). This result confirms the operability of the prototype under the selected parameter set and is consistent with the stable outlet-velocity regime identified by the CFD analysis. However, the measured rate is merely a verification result at the prototype level, rather than a quantitative validation of the CFD mass flow prediction. CFD-based pressure-drop predictions for extrusion-based printing have been reported to agree with experiments within approximately 13% at low discharge rates [22]; for the establishment of such quantitative comparisons based on mass flow rate for the current multi-chamber geometric structure, further research is still needed in the future. The resulting printed specimen is shown in Figure 18. The print quality demonstrates clear contour definition, tight interlayer bonding, and high dimensional accuracy, fully demonstrating that this nozzle can reliably achieve continuous, controlled material extrusion and effectively produce precisely shaped complex components, meeting the requirements for functional graded foam concrete 3D printing applications.

### 3.3. Functional Classification and Mechanical Properties of Foam Concrete

#### 3.3.1. Mechanical Properties

**Figure 19:**
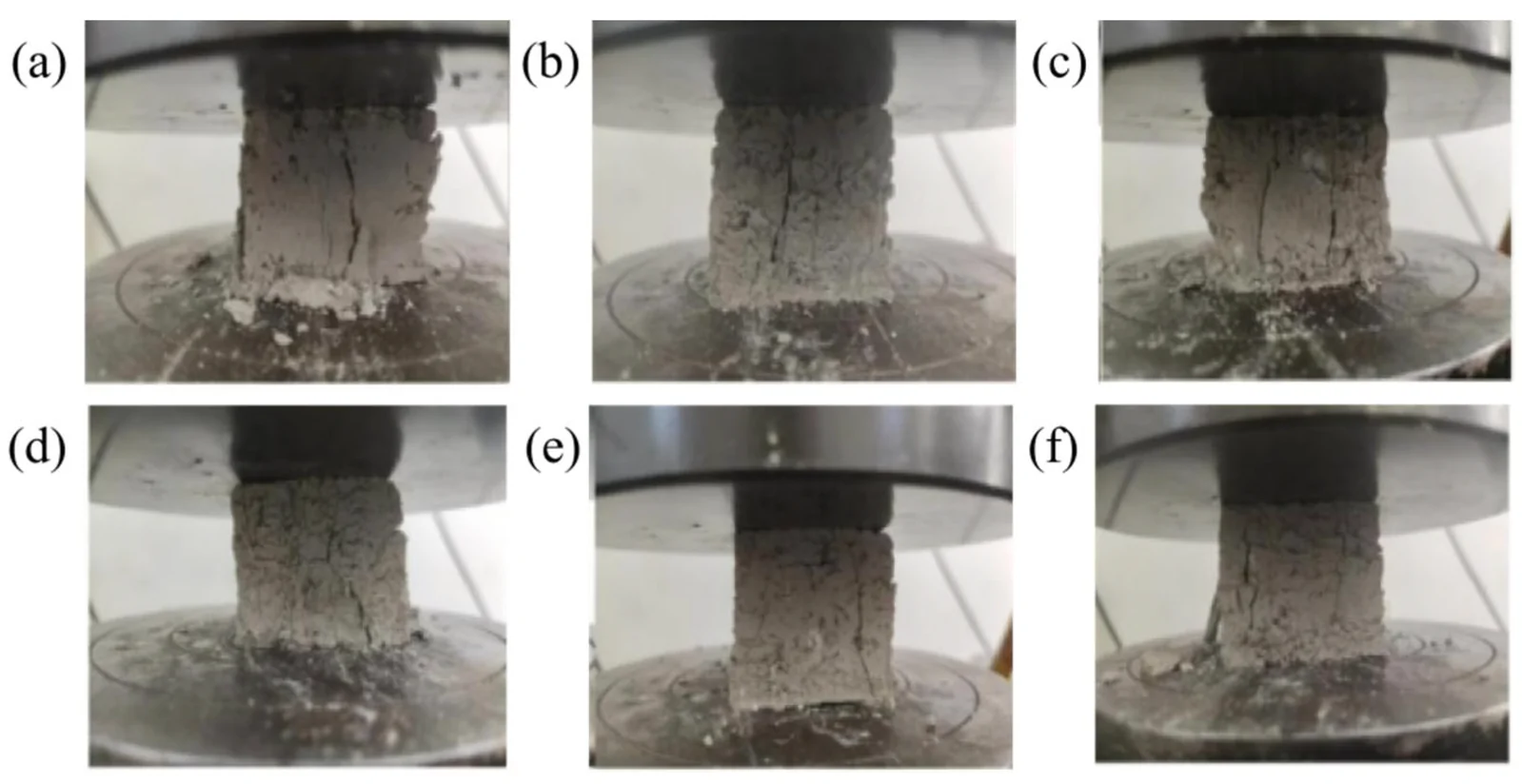
The compressive failure mode of functionally graded foam concrete: (**a**) H1; (**b**) H2; (**c**) H3; (**d**) H4; (**e**) H5; (**f**) H6.

**Figure 20:**
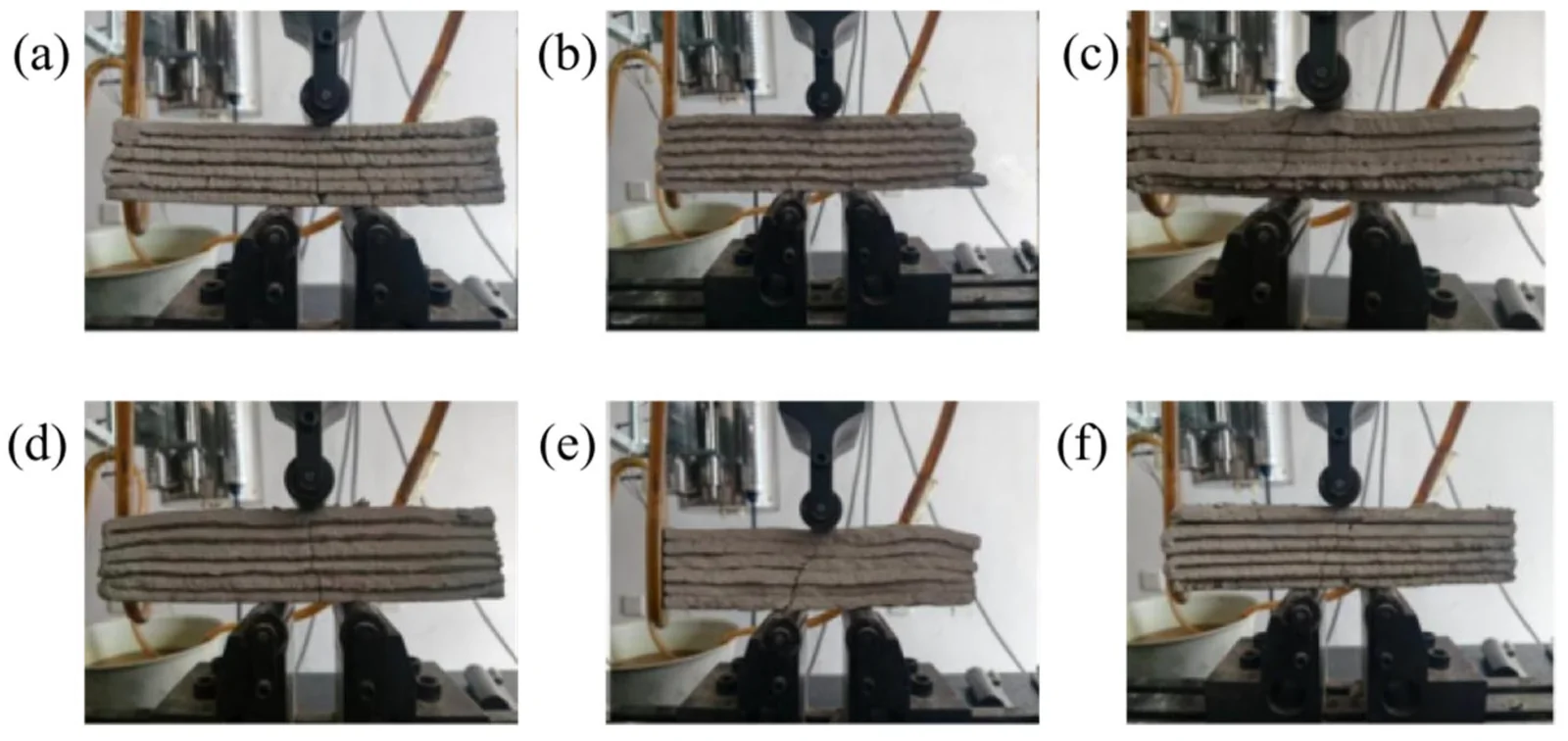
The flexural failure mode of functionally graded foam concrete: (**a**) H1; (**b**) H2; (**c**) H3; (**d**) H4; (**e**) H5; (**f**) H6.

**Figure 21:**
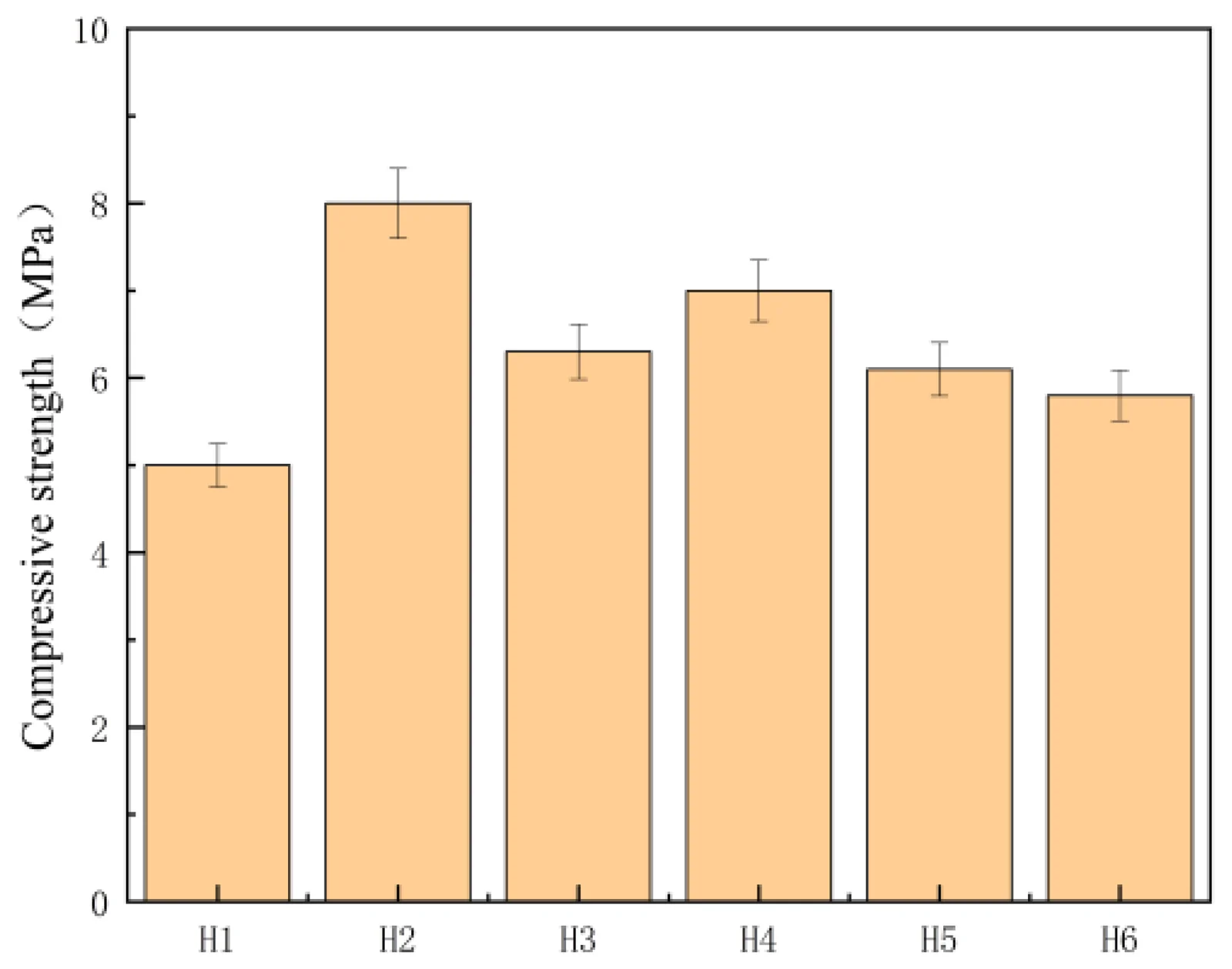
Bar chart of compressive strength of functionally graded foam concrete.

Figure 19 and Figure 20 respectively demonstrate the typical compressive and flexural failure morphologies of samples across groups. The compressive performance of functional gradient foam concrete is determined by both the layered arrangement and component composition, with the porous structure generated by a 40% fixed amount of foaming agent endowing the material with pore-structure-based thermal insulation potential. As shown in Figure 21, the compressive strength of H2 reaches 8 MPa, representing a 60% improvement over that of the homogeneous control group H1 (5 MPa). Data are means ± standard deviations of three specimens tested at a 28 d curing age.

In this gradient structure, the surface layer O1 incorporating 1% basalt fiber inhibits crack propagation through bridging effects, the middle layer O2 employs a 70% high-sand ratio to form a dense load-bearing framework, while the bottom layer O3 maintains a porous foam matrix, creating an integrated structure with toughened surface layers, load-bearing middle layers, and insulating base layers. For the flexural test, the simply supported span was 100 mm with the load applied from above. In the H2 specimen (O1-O2-O3 from bottom to top), the O1 layer containing 1% basalt fiber occupies the bottom tensile zone, O2 forms the middle load-transferring core, and O3 containing 40% foaming agent occupies the top compression zone. The H3 structure adopts the O1-O3-O2 layer sequence configuration, with the dense O2 layer located at the bottom. This layer sequence will cause a sudden change in stiffness, and compared to the H2 structure, it is expected to reduce the thermal insulation performance based on the pore structure. H5 and H6 position the fiber layer at the bottom, leading to premature cracking of the brittle matrix on the top surface and delayed crack inhibition effects, resulting in degraded mechanical and durability properties. Under fixed foaming conditions, H2 combines a fiber-reinforced O1 layer, a dense O2 core, and a porous O3 layer in an arrangement that is expected to favor the simultaneous retention of mechanical performance and pore-structure-based thermal-insulation potential, providing a reference for hierarchical design of multifunctional foam concrete systems. Subsequently, we will continue to conduct quantitative verification of the thermal conductivity of the H2 and H3 structures. The layer-sequence effect can be more directly assessed by comparing H2–H6, which have identical constituent materials but different layer sequences.

**Figure 22:**
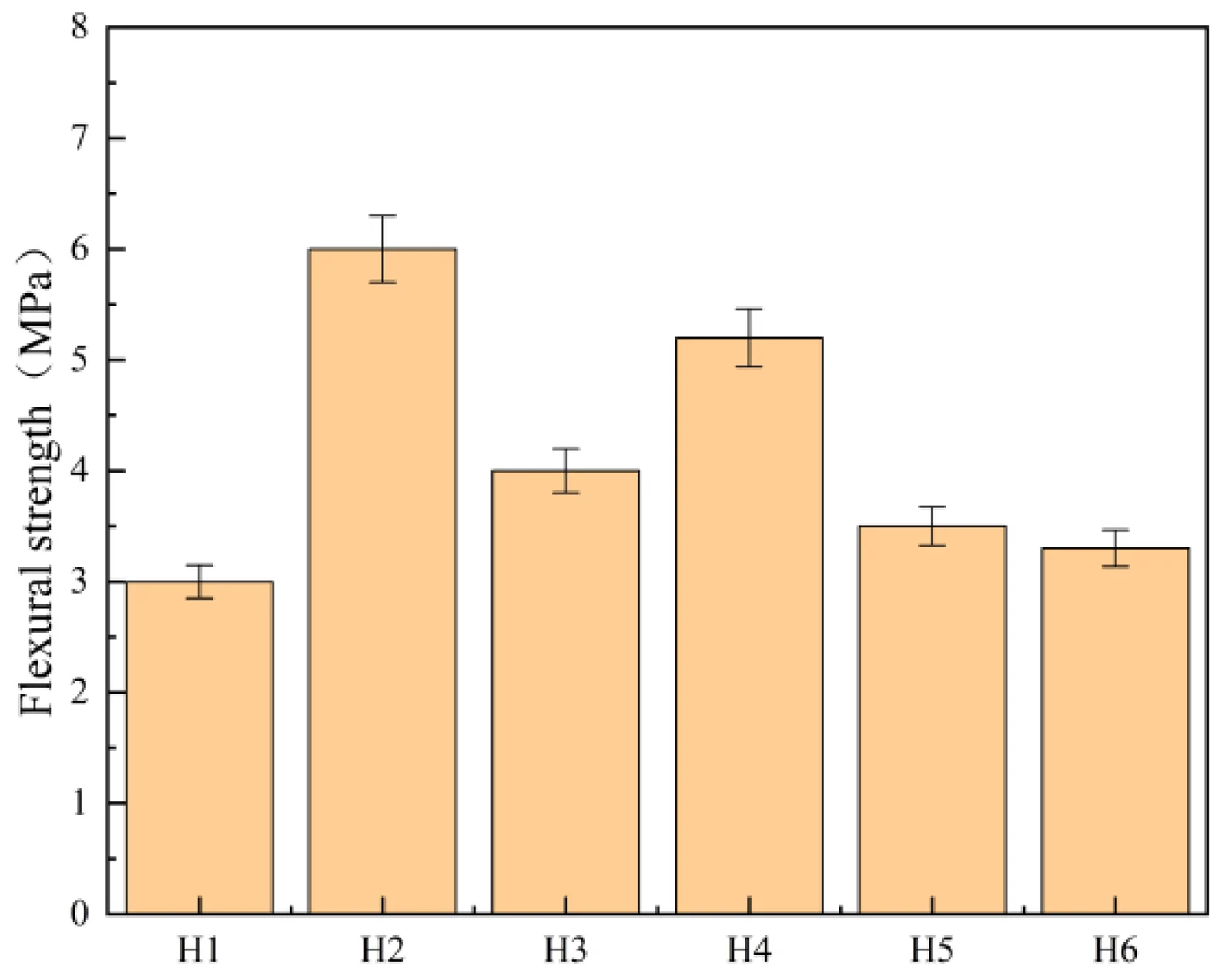
Stem chart of the flexural strength of functionally graded foam concrete.

As shown in Figure 22, the hierarchical configuration significantly regulates the flexural performance of specimens. The flexural strengths across all groups follow a clear hierarchy: H2 > H4 > H3 > H5 > H6 > H1, all exceeding that of the homogeneous sample H1. The peak flexural strength is 6 MPa for H2 and only 3.3 MPa for H6, reflecting differences in spatial arrangement effects across functional layers. At the component level, O1 incorporating 1% basalt fiber exhibits outstanding toughness and crack resistance, O2 with a high sand-to-cement ratio demonstrates dense structure and excellent load-bearing capacity, while O3 containing 40% foaming agent features abundant pores and the poorest flexural performance. H2 employs an O1-O2-O3 layered configuration—with high-toughness O1 in tension-receiving layers, dense O2 as an intermediate support layer, and low-strength insulating O3 at the top compression side—effectively aligning material properties with loading conditions and maximizing flexural performance through fiber–O3 synergy. In contrast, H6’s O3-O2-O1 arrangement places low-strength porous O3 in critical load zones while positioning high-strength O1/O2 in non-load-bearing areas, where numerous pores compromise matrix continuity and interfacial bonding, severely reducing flexural performance. Thus, rational hierarchical design is essential for enhancing gradient foam concrete’s flexural performance. Pairing high-strength components with stress-sensitive regions simultaneously achieves mechanical reinforcement and pore-structure-based thermal-insulation potential, fully leveraging the structural advantages of gradient configurations.

#### 3.3.2. Impact Resistance Analysis

**Figure 23:**
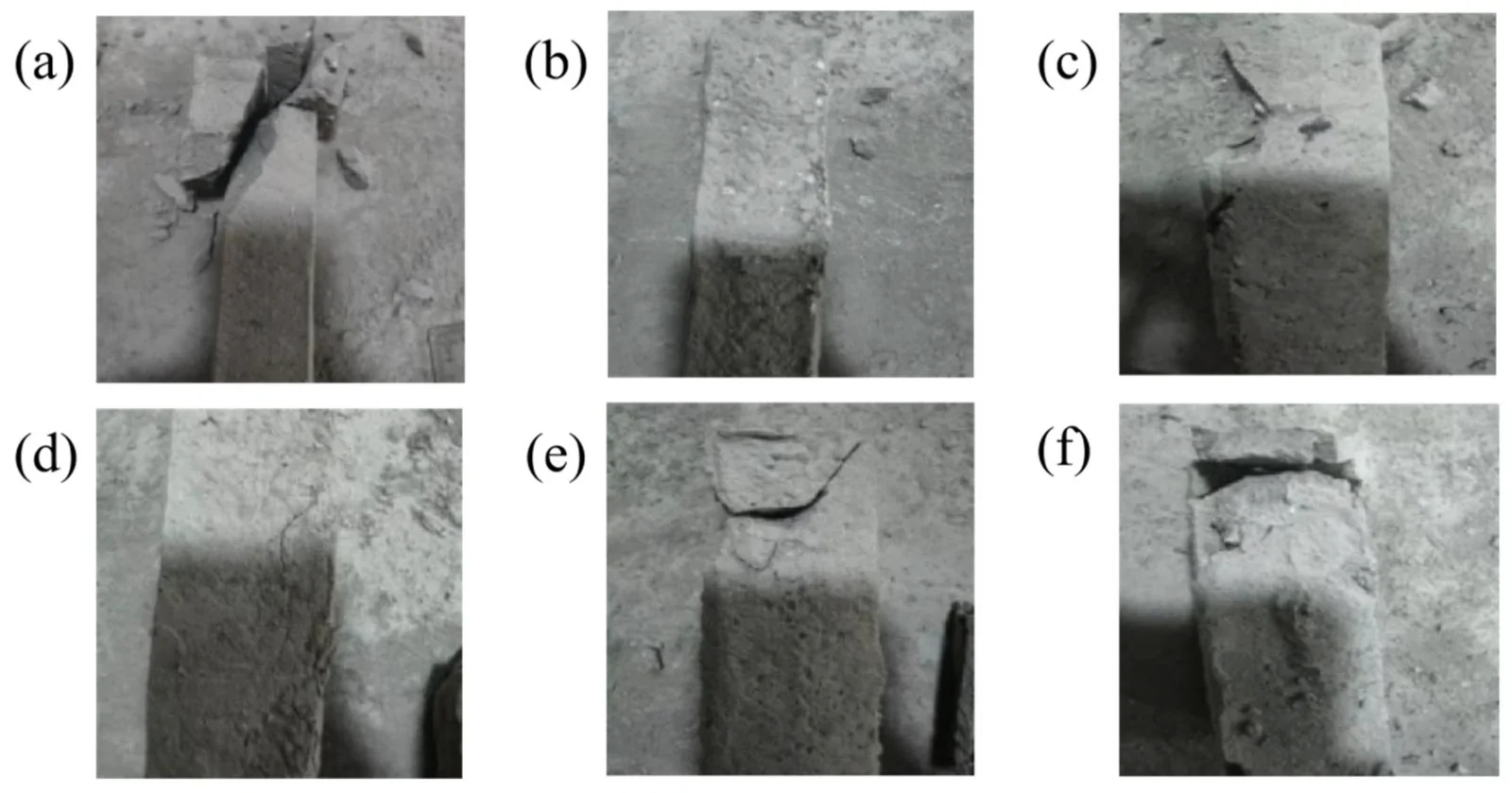
Impact damage pattern diagram: (**a**) H1; (**b**) H2; (**c**) H3; (**d**) H4; (**e**) H5; (**f**) H6.

As shown in Figure 23, the impact failure behavior of functionally graded foam concrete is highly dependent on its hierarchical structure. Quantitative drop-hammer impact tests (4.5 kg steel ball, 600 mm drop height, ACI 544) were performed for the homogeneous control H1 and the graded specimen H2. H2 required 12 ± 2 blows to first cracking and 28 ± 4 blows to failure, compared with 5 ± 1 and 15 ± 3 blows for H1 (3 specimens per group). These data support the following conclusion: compared with the homogeneous control group H1, the H2 sample demonstrates an enhanced resistance to repeated impacts. The homogeneous control group H1 exhibits an overall brittle fracture mode, attributable to the inherent brittleness of the pure foam matrix; groups H2–H6 demonstrate differentiated damage patterns due to interlayer stress redistribution, with H2 showing improved resistance to repeated impact relative to H1 as the surface fiber bridging interfaces effectively dissipate energy and suppress crack initiation; the middle layer O3 in H3 shows poor mechanical properties, leading to interfacial delamination and crack propagation along interlayer boundaries under impact; H4 and H5 exhibit similar failure modes but H4 demonstrates less damage, indicating that central placement of the fiber layer yields better crack inhibition than placement at the bottom; and in H6, cracks first initiate in the top layer O3, while the bottom layer O1 dissipates energy through fiber extraction, maintaining structural integrity. These results demonstrate that incorporating O1 fiber reinforcement layers in impact-resistant zones significantly enhances toughness, that gradient arrangement improves impact resistance by controlling crack propagation pathways, and that optimizing reinforcement composition and spatial distribution is critical for designing impact-resistant structures of such materials. It should be noted that greater resistance to repeated impacts does not automatically demonstrate greater ductility; the ductile-to-brittle transition claim is limited to the observed failure morphology.

## 4. Conclusions

Guided by the practical demand for lightweight and thermally insulating building materials in island and reef infrastructure under harsh marine environments, this study developed a multi-bin functionally graded 3D printing nozzle, optimized its key structural parameters through computational fluid dynamics simulation, and systematically evaluated the mechanical properties and impact resistance of functionally graded foam concrete prepared with the optimized equipment, thereby providing a preliminary technical basis for the intelligent on-site construction of island engineering.

(1) A novel printing nozzle composed of four independent auxiliary bins and one central main bin was designed, and its internal flow field was optimized through CFD simulation. The optimal screw pitches of the auxiliary and main bins and the spindle speed were determined as 15 mm, 30 mm, and 40 r/min, respectively, under which the chamber pressure remained stable and the outlet velocity stayed essentially constant, ensuring continuous and uniform extrusion. The independently driven chambers equipped with servo-controlled baffles and blade-type screw structures enable the separated transport of multi-component slurries and effectively minimize interlayer mixing during gradient printing. Continuous printing for 60 min yielded a stable extrusion rate of 833 g/min, which confirms the operability of the prototype under the selected parameter set.

(2) Three base matrices with differentiated functions, namely a basalt fiber-reinforced layer, a dense layer with a high sand-to-cement ratio, and a porous foam layer, were formulated, and six layer-sequence configurations were fabricated for comparison. Among all configurations, the O1-O2-O3 layered arrangement exhibited the best overall mechanical performance, increasing the compressive and flexural strengths by 60% and 100%, respectively, relative to the homogeneous control. This improvement arises from the synergistic mechanism in which the fiber-reinforced layer located in the tension zone suppresses crack propagation through fiber bridging, the dense middle layer serves as the load-transferring core, and the porous layer on the compression side preserves the pore-structure-based thermal insulation potential, indicating that aligning material functions with the stress distribution along the specimen thickness is decisive for the mechanical enhancement of functionally graded foam concrete.

(3) Quantitative drop-hammer impact tests were conducted on the homogeneous control and the optimal O1-O2-O3 graded specimen, and the graded specimen required 12 ± 2 blows to first cracking and 28 ± 4 blows to failure, compared with 5 ± 1 and 15 ± 3 blows for the homogeneous control, respectively, demonstrating that the rationally graded structure with the fiber-reinforced layer significantly improves the resistance to repeated impact loading through interfacial energy dissipation and crack-path control.

It should be emphasized that the study did not measure the quantitative thermal conductivity and the long-term marine exposure performance. It is suggested that these parameters be measured in future research to further verify the feasibility of the application of functionally graded 3D-printed foam concrete in island and reef engineering.

## Figures and Tables

**Table 1:** The chemical composition of silica fume.

Ingredient	SiO_2_	Al_2_O_3_	Fe_2_O_3_	CaO	MgO	K_2_O	Na_2_O
Content	90.02	1.03	0.60	0.72	3.02	3.05	1.51

**Table 2:** Function Gradient Layer Mix Ratio.

Group Number	Thialuminate Cement	Silica Fume	Fly Ash	River Sand	Water	Cellulose	Foaming Agent	Water Reducer	Basalt Fiber
O1	70%	5%	25%	42%	35%	2%	40%	3%	1%
O2	70%	5%	25%	70%	35%	2%	40%	3%	0%
O3	70%	5%	25%	42%	35%	2%	40%	3%	0%

Note: all percentages are mass percentages relative to the total mass of the cementitious binder (cement + silica fume + fly ash = 100%); the basalt-fibre content in O1 is a volume fraction of the concrete volume.

**Table 3:** The structural composition of the functional gradient foam concrete specimens.

Group Number	Number of Layers	Material Grouping	Material Grade Distribution (mm)
H1	6	O3	60
H2	6	O1, O2, O3	20-20-20
H3	6	O1, O3, O2	20-20-20
H4	6	O2, O1, O3	20-20-20
H5	6	O2, O3, O1	20-20-20
H6	6	O3, O2, O1	20-20-20

Note: the fiber volume fraction classification equals the difference between the fiber volume fractions of adjacent layers.

## Data Availability

The original contributions presented in this study are included in the article. Further inquiries can be directed to the corresponding authors.
